# Autonomous drug delivery and scar microenvironment remodeling using micromotor-driven microneedles for hypertrophic scars therapy

**DOI:** 10.1016/j.apsb.2025.05.017

**Published:** 2025-05-21

**Authors:** Ting Wen, Yanping Fu, Xiangting Yi, Ying Sun, Wanchen Zhao, Chaonan Shi, Ziyao Chang, Beibei Yang, Shuling Li, Chao Lu, Tingting Peng, Chuanbin Wu, Xin Pan, Guilan Quan

**Affiliations:** a School of Pharmaceutical Sciences, Sun Yat-sen University, Guangzhou 510006, China; b State Key Laboratory of Bioactive Molecules and Druggability Assessment, Guangdong Basic Research Center of Excellence for Natural Bioactive Molecules and Discovery of Innovative Drugs, College of Pharmacy, Jinan University, Guangzhou 510632, China; c Jiangmen Wuyi Hospital of Traditional Chinese Medicine, Affiliated Jiangmen Traditional Chinese Medicine Hospital of Jinan University, Jiangmen 529031, China

**Keywords:** Hypertrophic scar, Microneedles, Autonomous drug delivery, Drug diffusion, Autophagy regulation, Fibroblast apoptosis, Triamcinolone acetonide, Curcumin

## Abstract

Hypertrophic scar is a fibrous hyperplastic disorder that arises from skin injuries. The current therapeutic modalities are constrained by the dense and rigid scar tissue which impedes effective drug delivery. Additionally, insufficient autophagic activity in fibroblasts hinders their apoptosis, leading to excessive matrix deposition. Here, we developed an active microneedle (MN) system to overcome these challenges by integrating micromotor-driven drug delivery with autophagy regulation to remodel the scar microenvironment. Specifically, sodium bicarbonate and citric acid were introduced into the MNs as a built-in engine to generate CO_2_ bubbles, thereby enabling enhanced lateral and vertical drug diffusion into dense scar tissue. The system concurrently encapsulated curcumin (Cur), an autophagy activator, and triamcinolone acetonide (TA), synergistically inducing fibroblast apoptosis by upregulating autophagic activity. *In vitro* studies demonstrated that active MNs achieved efficient drug penetration within isolated scar tissue. The rabbit hypertrophic scar model revealed that TA-Cur MNs significantly reduced the scar elevation index, suppressed collagen I and transforming growth factor-*β*1 (TGF-*β*1) expression, and elevated LC3 protein levels. These findings highlight the potential of the active MN system as an efficacious platform for autonomous augmented drug delivery and autophagy-targeted therapy in fibrotic disorder treatments.

## Graphical abstract

An active microneedle (MN) system that employs citric acid (CA) and sodium bicarbonate (NaHCO_3_) as a built-in engine to generate CO_2_ bubbles, facilitating the combined delivery of triamcinolone (TA) and curcumin (Cur) to dense scar tissue and remodeling the scar microenvironment for effective hypertrophic scar treatment.

**Figure:**
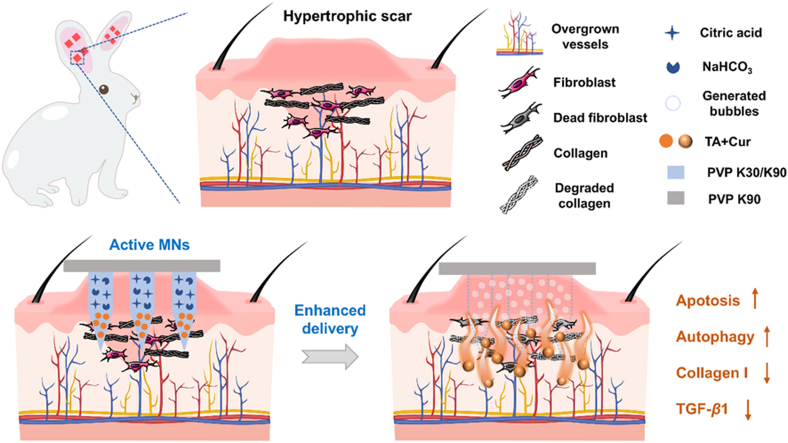

## 1 Introduction

Hypertrophic scar is a prevalent fibrous hyperplastic disorder that is characterized by aberrant fibroblast proliferation, excessive extracellular matrix (ECM) deposition, and disorganized collagen structure^1^. This condition typically arises as a consequence of pathological repair mechanisms following injuries to the skin, such as trauma, burns, or surgical wounds. In clinical practice, hypertrophic scars typically manifest as elevated, firm, red plaques that cause discomfort, including itching and tingling, and often give rise to considerable physical, psychological, and cosmetic concerns for patients^2^.

The current treatment strategies for hypertrophic scar encompass surgical, physical, and pharmacological approaches^3^. However, these treatments have several limitations, such as the high recurrence rate of surgical excision^4^, slow onset and prolonged use of compression therapy^5^, and high cost of laser therapy^6^. In light of these challenges, pharmacotherapy is frequently the preferred option due to its accessibility and broader applicability, with corticosteroids^7^, particularly intralesional triamcinolone acetonide (TA) injections, are widely recommended. TA exerts its effect by inhibiting fibroblast growth and collagen synthesis^8^. However, high-dose TA treatment can result in adverse effects, including skin atrophy, capillary dilation, and pigmentation disorders, which restrict its application^9^^,^^10^. Consequently, alternative therapeutic options are urgently required to enhance treatment efficacy and safety.

The pathogenesis of hypertrophic scarring is an intricate process involving multiple mediators in the wound healing cascade^11^, many of which remain to be fully elucidated. Autophagy, a conserved cellular mechanism that regulates intracellular turnover, plays a critical role in maintaining cellular homeostasis^12^. During autophagy, damaged organelles or proteins are engulfed and wrapped in autophagosomes, which then fuse with lysosomes to degrade and recycle their contents^13^^,^^14^. Insufficient autophagy impedes the completion of the regressive healing phase in hypertrophic scarring, resulting in limited autophagy-mediated apoptosis and elevated matrix deposition^15^. Indeed, the expression of autophagy-related proteins, such as LC3-II and Beclin1, is markedly diminished in hypertrophic scar tissue compared to normal skin tissue^16^. Augmenting autophagy may potentially induce cell apoptosis and facilitate matrix regression, thereby providing a promising therapeutic avenue for attenuating hypertrophic scarring.

The administration of pharmaceutical agents to hypertrophic scar tissue is typically achieved *via* direct multi-point injections. However, this approach presents several drawbacks. The dense, hardened scar structure often results in drug leakage from injection sites, which in turn leads to poor dose accuracy and uneven drug distribution. Furthermore, passive diffusion is ineffective for deep dermal penetration and conventional injection results in substantial pain, which reduces patient compliance and potentially causes local side effects, such as skin atrophy, hypertension, and osteoporosis. Recently, microneedles (MNs) have emerged as a promising, minimally invasive option for transdermal drug delivery^17^^,^^18^. Compared to traditional transdermal preparations such as ointment, MNs can overcome stratum corneum barrier, facilitating efficient drug delivery to deeper skin layers^17^^,^^19^^,^^20^. However, traditional dissolving MNs rely on passive diffusion, which is often inadequate for penetrating the dense structure of hypertrophic scar tissue.

**Scheme 1:**
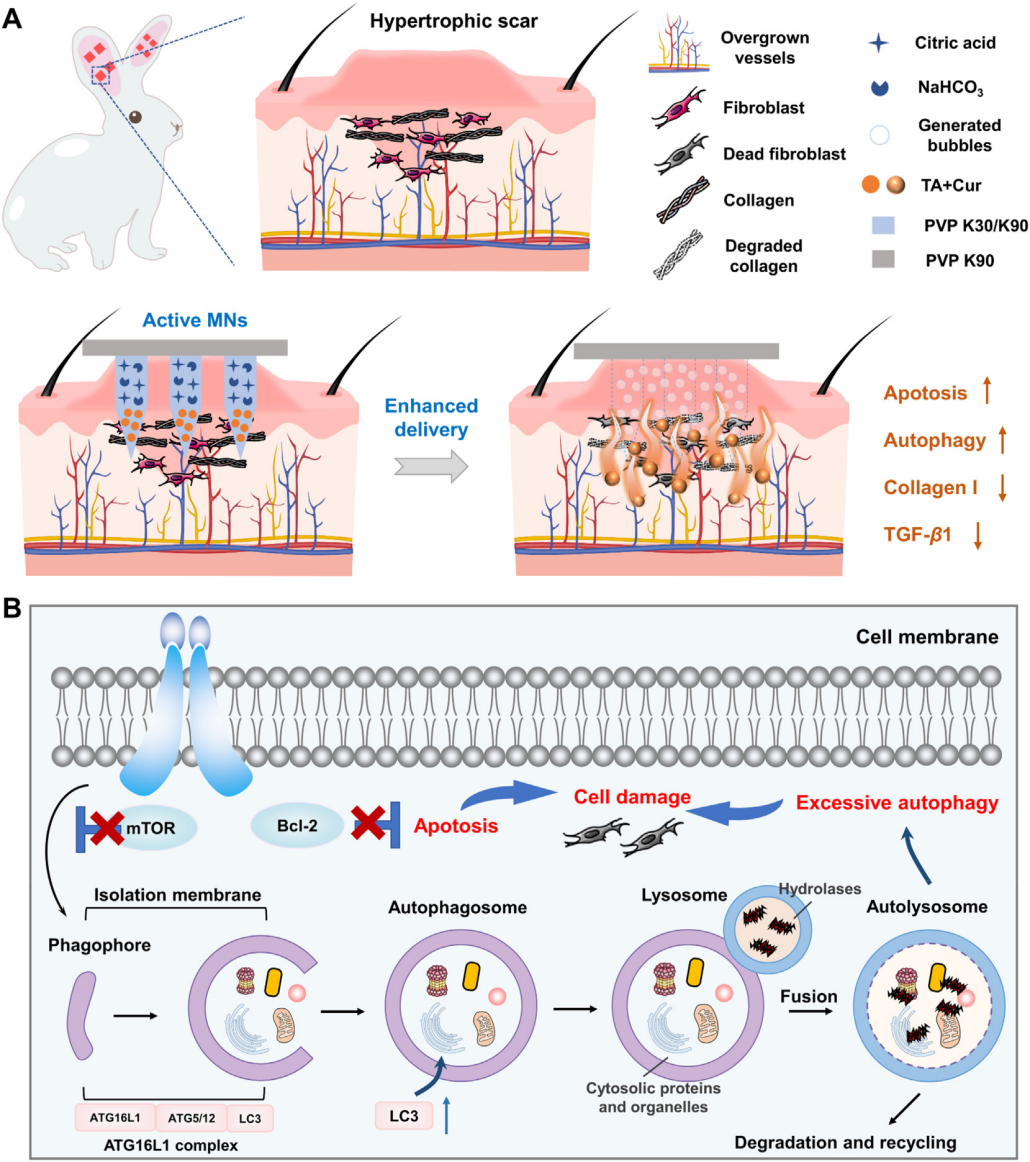
Schematic illustration of active MNs for hypertrophic scar therapy. (A) The process of active MNs for enhanced drug delivery against hypertrophic scar, and (B) their mechanisms of inducing autophagy and apoptosis in scar fibroblasts.

To address these challenges, we developed an innovative active MN system with micromotor-driven drug diffusion for enhanced deep penetration in dense hypertrophic scars. The system employs a co-loading approach, whereby TA and curcumin (Cur) are incorporated into polyvinylpyrrolidone (PVP)-based MNs. Cur was explored as a potential autophagy activator with the objective of reshaping the scar microenvironment^21^^,^^22^. Upon application, the MNs undergo dissolving, releasing a pneumatic layer composed of citric acid and sodium bicarbonate (NaHCO_3_) microparticles, which react to generate CO_2_ bubbles. These bubbles facilitate rapid MN separation from the patch backing, thereby minimizing wear time, and create vortex flow fields that act as “pumps”, enhancing three-dimensional diffusion and penetration of TA and Cur into the deep lesion (Scheme 1A).

This delivery method achieves localized and deep intracutaneous distribution of TA and Cur, where Cur synergizes with TA by promoting fibroblast apoptosis through autophagy activation. In a rabbit ear hypertrophic scar model, this system effectively repaired scar tissue, underscoring the therapeutic potential of autophagy induction in treating hypertrophic scars (Scheme 1B). Overall, this active MN system offers valuable insights into efficient, autonomously enhanced drug delivery and highlights the promising role of autophagy in scar management.

## 2 Materials and methods

### 2.1 Materials

TA was purchased from Jieshikang Biotechnology Co., Ltd. (Qingdao, China). Cur, rapamycin (Rapa), chloroquine phosphate (CQ), 3-methyladenine (3-MA), NaHCO_3_, gelation (adhesive strength ∼240 g), coumarin 6 (C6), and Cy5.5 were all provided by Aladdin Biochemical Technology Co., Ltd. (Shanghai, China). Citric acid was obtained from Damao Chemical Reagent Co., Ltd. (Tianjin, China). Human skin fibroblast (HSFs) were provided by Bioleaf Biotechnology Co., Ltd. (Shanghai, China). Trypsin (EDTA free) was purchased from Roles-Bio Biotechnology Co., Ltd. (Guangzhou, China), and cell counting kit-8 (CCK-8) was provided by DIB Data Inventory Biotechnology (Guangzhou, China). Diamidinophenyl indole (DAPI) staining solution and 4% paraformaldehyde were purchased from Biosharp Biotechnology Co., Ltd. (Beijing, China). PVP K30 and PVP K90 were kindly donated by BASF SE (Ludwigshafen, Germany). All reagents were used without further purification.

### 2.2 Cell proliferation inhibition assay

To identify the optimal ratio of TA to Cur, the concentration of Cur was fixed at 10 μmol/L and the concentrations of TA were adjusted accordingly. HSFs were inoculated in 96-well plates overnight, and then 100 μL of the TA-Cur mixed solution was added to each well. After 24 h of incubation, the cell viability was determined using CCK-8 assay.

### 2.3 Cell wound scratch assay

The HSFs were inoculated in 6-well plates (7 × 10^5^ cells per well). On the subsequent day, a sterile pipette tip was employed to create a scratch in the center of each well, and PBS was gently added to rinse the floating cells. The remaining cells were subjected to different treatments: Control, TA, Cur, and TA + Cur. Photographs of the scratch were recorded at 0, 12, 24, and 48 h. The scratch healing rate was calculated according to Eq. (1), where *A*_0_ and *A*_*t*_ represent the scratch area at 0 and *t* h, respectively.

(1) Scratch healing rate (%) = (*A*_0_ – *A*_*t*_) / *A*_0_ × 100

### 2.4 Live/dead cell staining assay

The HSFs were inoculated at a density of 3 × 10^5^ cells per confocal dish and incubated for 24 h. Then the cells were subjected to various treatments for 24 h, including Control, TA, Cur, and TA + Cur. The Calcein AM/PI test solution (Shanghai Beyotime Biotechnology Co., Ltd.) was then added and incubated for 30 min at 37 °C in the dark. After washing with PBS, the cells were observed under a confocal laser scanning microscope (CLSM, FV3000, Olympus Corporation, Japan).

### 2.5 Cell apoptosis detection

The HSFs were inoculated in 6-well plates at a density of 7 × 10^5^ cells per well and incubated for 24 h, and then treated with different drug solutions for another 24 h. The cells were harvested by digestion and centrifugation at 120×*g* for 5 min. Cell pellets were re-suspended in pre-cooled PBS and centrifuged again to remove any residual dye. Binding buffer containing 5 μL of Annexin V-FITC and 10 μL of PI was added to stain the cells for 15 min. Finally, the samples were diluted with 400 μL of PBS and detected by flow cytometry.

### 2.6 Cur-induced HSF cell death patterns

To ascertain the patterns of cell death induced by Cur, different inhibitors were added to co-incubate with Cur and HSFs, including Z-VAD-FMK (apoptosis inhibitor), Necrostatin-1 (necrosis inhibitor), and Ferrostatin-1 (Fer-1, ferroptosis inhibitor). Besides, the autophagy promoter Rapa and autophagy inhibitors (CQ and 3-MA) were applied to investigate the correlation between Cur-caused cytotoxicity and autophagy. The cytotoxicity was quantified as previously described.

### 2.7 Detection of Cur-induced autophagy in HSFs

The formation of autophagic vesicles was assessed using Monodansylcadaverin (MDC) and LysoTracker staining^23^^,^^24^. HSFs were seeded at a density of 3 × 10^5^ cells per confocal dish and cultured overnight. Then, the cells were treated with Control, Cur, Cur + Rapa, Cur + CQ, or Cur+3-MA for 4 or 24 h. Following staining, the cells were imaged using CLSM.

### 2.8 Cherry-eRFP-LC3 plasmid transfection

Double-labeled LC3 transfection technique was used to monitor the autophagy levels^25^. The HSFs (2.5 × 10^5^ cells) were transfected with an mCherry-eGFP-LC3 plasmid (2 μg) using jetPRIME® buffer to form a DNA-jetPRIME® complex. The obtained mCherry-eRFP-LC3-expressing HSFs were incubated with different samples for 4 or 24 h, fixed with 4% paraformaldehyde for 30 min, stained with DAPI solution for 15 min, and the expressions of LC3-I and LC3-II proteins were observed under CLSM.

### 2.9 Observation of autophagic vacuoles by biological TEM

The HSFs (1.5 × 10^6^/dish) were inoculated overnight and treated with different samples for 24 h. The cells were washed with PBS, fixed with 2.5% glutaraldehyde fixative for 5 min, scraped off, centrifuged at 265×*g* for 3 min, and stored overnight at 4 °C. After additional processing, samples were observed using a transmission electron microscope (TEM, H-7650, Hitachi, Japan).

### 2.10 Detection of related proteins by Western blot

The treated cells were collected and the concentration of total proteins was determined by BCA method. The proteins were separated by sodium dodecyl sulfate-polyacrylamide gel electrophoresis (SDS-PAGE), transferred onto polyvinylidene fluoride (PVDF) membranes, and blocked with 5% skim milk. The obtained membranes were incubated with primary antibody overnight at 4 °C, followed by secondary antibody incubation for 1 h at room temperature. Finally, the membrane was visualized using a chemiluminescence imaging system (QuickChemi 5100, Suzhou Monad Biotechnology Co., Ltd.).

### 2.11 Preparation of TA-Cur loaded MNs

The TA-Cur active MNs were fabricated through a multi-step centrifugation method. Briefly, Cur and TA with a molar ratio of 1:2 were dissolved in anhydrous ethanol, and mixed with 250 mg/mL PVP K30/PVP K90 ethanol solution (PVP K30: PVP K90 = 1:1, *w*:*w*) at a volume ratio of 4:1, which was spread onto the female mold and centrifugated at 1800×*g* for 5 min under 4 °C. After removal of the excess solution, the female mold was dried overnight in a desiccator. On the subsequent day, the pneumatic layer solution consisting of 4% citric acid and 5% NaHCO_3_ in the PVP K30/PVP K90 ethanol solution (250 mg/mL) was added under similar conditions. Finally, PVP K90 ethanol solution (*w*/*v*, 1:3.2) was used to constitute the backing part. After drying for 2 days, TA-Cur active MNs were gently peeled off the female mold. For comparison, TA-Cur passive MNs, blank active MNs, TA active MNs, and Cur active MNs were prepared similarly.

### 2.12 Morphology and element analysis of active MNs

Scanning electron microscope (SEM, EVO MA10, Shanghai Carl Zeiss Management Co., Ltd., China) was employed to observe the morphology of MNs. The energy dispersive X-ray spectroscopy (EDX) was used to analyze the element distribution. Besides, the fluorescence of Cur was utilized to reconstruct the 3D morphology under CLSM.

### 2.13 Skin puncture ability of active MNs

Trypan blue-loaded MNs were prepared and applied to the shaved rat skin for 2 min, after which the resulting micropores on the skin were photographed. Hematoxylin–eosin (H&E) staining and SEM were used to evaluate the puncture and dissolution properties.

### 2.14 Gas generation capacity of active MNs

A piece of MNs was fixed to a Petri dish with double-sided adhesive, and then the aqueous solution was sucked to immerse the patch. The gas generation and dissolution of MNs were recorded with a handheld microscope.

### 2.15 Dissolution in gelatin-simulated skin of active MNs

MNs were vertically inserted into gelatin blocks containing 35% water to simulate their dissolution in skin tissue. After 4 min, the patch was carefully removed and placed on an inverted microscope to observe the surface of gelatin blocks and patch backing.

### 2.16 Drug diffusion and penetration delivered by MNs

To visualize the drug delivery behavior, MNs labelled with C6 and Cy5.5 were inserted into the isolated rat skin and observed using CLSM. The longitudinal distribution and penetration behavior were also evaluated in isolated scar tissues. The samples subjected to MNs was quickly frozen in liquid nitrogen and then transferred to a −80 °C refrigerator followed by frozen sectioning and observation.

### 2.17 *In vitro* drug release in MNs

The needle tips of MNs were excised and collected into a centrifuge tube containing 7 mL of release medium (PBS, pH 5.8–6.0), followed by shaking at a speed of 100 rpm under 37 °C. At predetermined time points, the receiving solution was sampled and replaced with fresh medium. Following filtration by a 0.22 μm membrane, the drug release amounts from MNs were determined by HPLC.

### 2.18 *In vivo* drug retention and distribution

The *in vivo* drug retention and distribution of MNs were investigated in C57BL/6 female mice. A piece of passive or active MNs was applied to the dorsal skin of mouse, and the fluorescence intensity was detected at different time points by a small animal imager (NightOWL Ⅱ LB983, BERTHOLD, Germany). After 24 h, the local skin and main organs (heart, liver, spleen, lung, and kidney) were collected for observation of residual fluorescence intensity.

### 2.19 Establishment of hypertrophic scar model

To assess the *in vivo* therapeutic efficacy of TA-Cur active MNs, a hypertrophic scar model was established on the rabbit ears as previously reported^26^, ^27^, ^28^. Briefly, New Zealand rabbits weighing 1.8–2.3 kg were anesthetized, and four wounds (1 cm × 1 cm) were created on the ventral side of each ear. The full-thickness skin and perichondrium within the wounds were excised to expose the cartilage surface. The newly formed scabs were removed to expose the wounds until healing was completed. Following the excision of the skin for 21 days, the hypertrophic scar model was successfully constructed with a thickness ratio of scar skin to normal skin greater than 1.5. All the animal experimental procedures above were conducted with the approval of the Animal Ethics Committee of Sun Yat-sen University (Approval No. SYSU-IACUC-2022- 001302).

### 2.20 Administration regimen

The hypertrophic scar-bearing rabbits were randomly divided into 7 experimental groups, with normal rabbits serving as controls. The specific groups were as follows: (G1) normal skin, (G2) hypertrophic scar (negative control), (G3) TA-Cur injection (positive control), (G4) blank active MNs, (G5) TA active MNs, (G6) Cur active MNs, (G7) TA-Cur passive MNs, (G8) TA-Cur active MNs. No treatment was administered to groups G1 and G2. The TA-Cur injection group received a mixed injection of TA and Cur, prepared according to the clinical administration protocol. The amount of drug injected was identical to that of MNs. For the MN-treated groups, the MN patches were applied vertically to the scar for 2 min and then fixed with pressure-sensitive adhesive. The administrations were performed on Days 22, 29, and 36 post-surgery. One week following each administration (*i.e.*, Days 29, 36, and 43 post-surgery), the surface appearance of scar wounds was observed and photographed. The scar tissues were collected for further histological analysis and protein expression determination.

### 2.21 Histological staining and analysis

The extracted skin tissue was fixed in 4% paraformaldehyde for 24 h, embedded in paraffin, and sliced. Subsequently, H&E, Masson, and Sirius red staining were conducted to evaluate the skin re-epithelialization, collagen deposition, and conformational changes of collagen fibers, respectively. The Image J software was used to analyze the H&E tissue sections, and the Scar elevation index (SEI) was calculated as Eq. (2):

(2) SEI = *H* / *H*_0_

where *H*_0_ represents the distance between the surrounding normal skin stratum corneum to the cartilage surface, and *H* represents the distance between the thickest stratum corneum and the cartilage surface in the hypertrophic scar.

### 2.22 Protein expression analysis

The expression of related proteins was assessed using immunohistochemical (IHC) staining and Western blot analysis. For IHC staining, paraffin-embedded sections were prepared and then incubated sequentially with primary and secondary antibodies. For Western blot analysis, the samples stored at −80 °C were retrieved, the cartilage tissues were removed, and the samples were minced and placed in tissue homogenate tubes with lysis buffer to obtain homogenates. After centrifugation at 17,500×*g* for 5 min under 4 °C, the supernatants were collected and placed on ice for protein quantification using a BCA kit. The remaining steps were conducted as previously described.

### 2.23 Statistic analysis

All data are presented as mean ± standard deviation (SD). Statistical analyses were conducted through *t*-test or one-way analysis of variance (ANOVA) using Graphpad Prism 9 (Graphpad Software LLC., San Diego, CA, USA). *P* value < 0.05 was considered statistically significant.

## 3 Results and discussion

### 3.1 Proliferation inhibition of HSFs

#### 3.1.1 Cytotoxicity of TA and Cur

**Figure 1:**
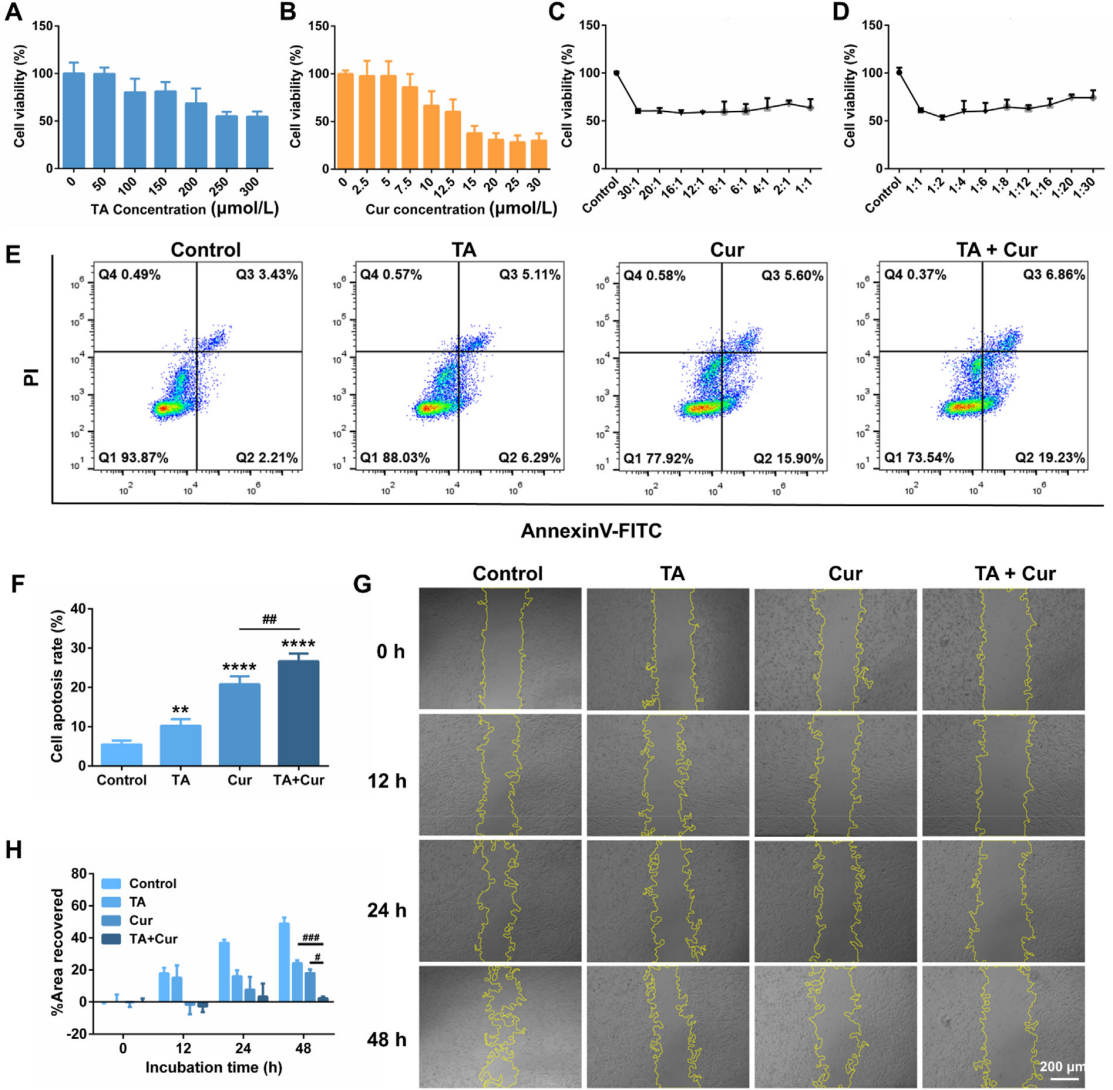
Apoptosis and migration assay of HSFs by different treatments. Viability of HSFs treated by (A) TA and (B) Cur solutions (*n* = 6). (C, D) Viability of HSFs after treated with Cur in conjunction with TA at different molar ratios (*n* = 3). (E) Apoptosis and necrosis analysis of HSFs after different treatments. (F) Statistics analysis of HSF cell apoptosis rate (*n* = 4). Note: ∗∗*P* < 0.01 *vs* control, ∗∗∗∗*P* < 0.0001 *vs* control, ^##^*P* < 0.01 *vs* TA + Cur. (G) The HSF cell migration in scratch assay after receiving different treatments (scale bar: 200 μm). (H) The percentage of the recovered area after various treatments (*n* = 3). Note: ^#^*P* < 0.05, ^###^*P* < 0.001.

Serial concentrations of TA and Cur were employed to incubate with HSFs for 24 h, and their viabilities were determined. As illustrated in Fig. 1A, the viability of HSFs was not distinctly impacted at low concentrations of TA. Upon elevating the TA concentration to 250 μmol/L, 55.1% of cells remained viable. The administration of Cur alone resulted in a viability of approximately 66.5% for HSFs at a molar concentration of 10 μmol/L (Fig. 1B). These results demonstrate that TA exerts a pronounced inhibitory effect on HSFs at elevated concentrations, as TA belongs to the adrenocortical hormone class and primarily functions as an anti-inflammatory agent^27^. Conversely, low concentrations of Cur exhibited a strong inhibitory impact on the proliferation of HSFs.

The impact of TA in combination with Cur on HSF proliferation was further explored. The molar concentration of Cur was fixed at 10 μmol/L, while the molar ratio of Cur to TA was varied from 30:1 to 1:30. As displayed in Fig. 1C and D, the cell survival rate was 60.4% and 74.2%, respectively, when the Cur/TA molar ratio was 30:1 and 1:30. The lowest cell viability of 53.4% was observed under the ratio of 1:2, which exhibited the most pronounced decline compared to 66.5% induced by Cur alone at the same concentration. Besides, the TA concentration in this case was only 20 μmol/L, which was considerably higher than the effect of TA alone at this concentration. This suggests that the combined ratio was optimal and could be used for subsequent studies.

#### 3.1.2 Cell apoptosis

The detection of cell apoptosis and necrosis was conducted using the Annexin V-FITC/PI detection kit. In the case of early apoptotic cells, the transfer of phosphatidylserine from the inner to the outer surface of the cell membrane occurs, where it binds to the phospholipid-binding protein Annexin-V. PI is capable of penetrating the damaged cell membranes of late apoptotic and necrotic cells, in contrast to the intact cell membranes of normal and early apoptotic cells. As illustrated in Fig. 1E and F, the apoptosis rates of the four groups (Control, TA, Cur, TA + Cur) were 5.4%, 10.2%, 20.8%, and 26.6%, respectively. The necrosis rate was less than 1%, and thus could be considered negligible. In conclusion, the combined treatment of TA and Cur was observed to induce apoptosis in HSFs, predominantly early apoptosis, which subsequently facilitated the further inhibition of cell growth and proliferation.

#### 3.1.3 Live/dead cell staining

To further evaluate the effects of monotherapy and combined treatment on HSFs, a Calcein/AM/PI staining procedure was employed to differentiate between live and dead HSFs. As shown in Supporting Information Fig. S1, the control group exhibited dense, well-shaped, and intensely green fluorescent HSFs. Following incubation with TA alone, the cell morphology exhibited slight alterations, while the majority of HSFs displayed morphological atrophy with a weak PI red fluorescence following treatment with Cur. When the cells were subjected to the combined administration of TA and Cur, the green fluorescence density was markedly diminished, and the nuclei underwent shrinkage without complete cell morphology, while the red fluorescence density increased significantly. The combined administration of TA and Cur was demonstrated to present a more pronounced induction effect on cell death and to be superior to monotherapy.

#### 3.1.4 *In vitro* cell migration

The excessive proliferation of scar fibroblasts and their strong migration ability represent primary factors contributing to the formation of hypertrophic scar. The cell scratch method allows for the simulation of the migration, movement, and repair abilities of fibroblasts. As illustrated in Fig. 1G and H, the HSFs in the control group began to migrate to the center of the scratch area at the 12-h time point, and the healing area reached 49.0% at 48 h. When TA was administered alone, the HSF cell migration ability was inhibited to a certain extent. The extent of healing of HSFs was about 24.2% after 48 h. Likewise, the healing rate reached 18.0% at 48 h for the Cur-treated group, indicating that both TA and Cur had a certain inhibitory effect on cell migration, but the influence of Cur was more pronounced. Following 48 h of combined administration of TA and Cur, the cell healing rate was only 2.4%, indicating that TA in conjunction with Cur exerted a pronounced inhibitory effect on HSF cell migration.

### 3.2 Analysis of Cur-induced HSF cell death modes

**Figure 2:**
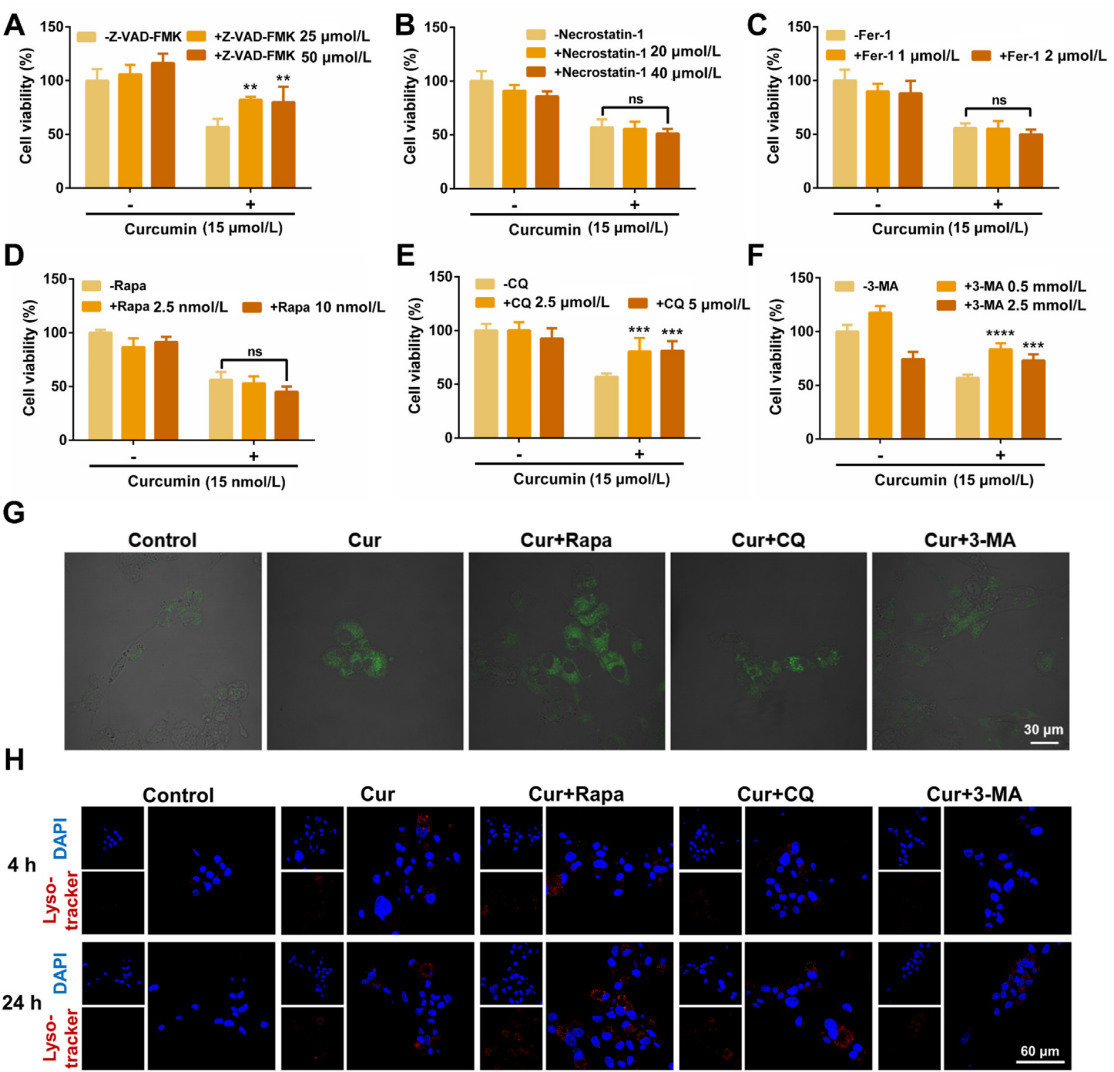
Analysis of death forms of HSFs after different treatments. Effects of (A) Z-VAD-FMK, (B) Necrostatin-1, (C) Fer-1, (D) Rapa, (E) CQ, and (F) 3-MA on Cur-induced HSF cytotoxicity (*n* = 6). Note: ns signified no significant difference, ∗∗*P* < 0.01 *vs* Cur, ∗∗∗*P* < 0.001 *vs* Cur, ∗∗∗∗*P* < 0.0001 *vs* Cur. Change of autophagic vesicles in HSFs detected by (G) MDC (scale bar: 30 μm) and (H) Lyso-tracker (scale bar: 60 μm).

To analyze the form of HSF cell death induced by Cur, three independent inhibitors were added: Z-VAD-FMK (apoptosis inhibitor), necrostatin-1 (necrosis inhibitor), and Fer-1 (ferroptosis inhibitor). As illustrated in Fig. 2A–C, in the absence of inhibitors, the viability of HSFs was approximately 56.0% following treatment with Cur at 15 μmol/L. However, this rate increased to 82.2% and 80.0%, respectively, following co-incubation with 25 or 50 μmol/L of Z-VAD-FMK (Fig. 2A). These results indicate that apoptosis inhibitors can effectively mitigate the cytotoxic effect of Cur on HSFs (∗∗*P* < 0.01). Following co-incubation with 20 or 40 μmol/L of necrostatin-1, the viabilities of HSFs were 55.6% and 51.2%, respectively (Fig. 2B). Similarly, the cell viabilities were 55.3% and 49.9%, respectively, following co-incubation with 1 or 2 μmol/L of Fer-1 (Fig. 2C). Therefore, it was speculated that Cur promoted HSF cell death *via* the apoptosis-related pathway, rather than necrosis or ferroptosis, which was also consistent with the aforementioned results obtained by Annexin V-FITC/PI apoptosis assay.

To further explore the potential relationship between Cur-induced HSF cell death and the autophagy pathway, the classical autophagy promoter (Rapa) and autophagy inhibitors (CQ and 3-MA) were employed to ascertain their impact on the cytotoxicity of Cur. As presented in Fig. 2D, the cell viability exhibited a slight decline following the co-treatment with 2.5 or 5 nmol/L of Rapa. This suggests that the combined treatment may have an enhanced effect, although no statistically significant difference was observed in comparison to Cur alone. The combination of Cur with 2.5 or 5 μmol/L of CQ resulted in an increase in cell viability to 80.5% and 81.0%, respectively (Fig. 2E). Furthermore, the cell viability was also observed to increase when the cells were treated with 3-MA (Fig. 2F). Thus, the results demonstrated that autophagy inhibitors significantly suppressed the cytotoxicity of Cur, indicating that the HSF cell death induced by Cur was associated with its autophagy-promoting effect.

### 3.3 Cur-induced autophagy in HSFs

#### 3.3.1 Detection of autophagic vesicles

Autophagy is a process whereby cells engulf cytoplasmic proteins or organelles and envelope them in vesicles to form autophagosomes. These are subsequently fused with lysosomes to form autolysosomes, which degrade the contents of the vehicles. MDC staining was firstly employed to label acidic autophagic vesicles. As shown in Fig. 2G, the MDC fluorescence was observed to be enhanced in the Cur-treated group, suggesting an increase in the number of acidic vesicle organelles and indicating that Cur has the potential to promote autophagy. Following co-treatment with Rapa, the green fluorescence was also strengthened, yet no significant discrepancy was observed in the semi-quantitative results (Supporting Information Fig. S2). The fluorescence intensity difference between the Cur + CQ and Cur groups was not significant, as CQ neutralizes H^+^ in the autolysosomes, increasing pH and weakening lysosomal degradation. This results in the accumulation of autophagosomes in cells, ultimately blocking the autophagy process^25^. Therefore, CQ was observed to induce a significant accumulation of acidic vesicle organelles. In comparison to the Cur group, the Cur+3-MA group exhibited impaired fluorescence (^#^*P* < 0.05). The mechanism of action of 3-MA is primarily to inhibit the formation and development of autophagic vacuoles, which suggests that 3-MA may have inhibited the autophagy-promoting effect of Cur, resulting in a reduction in autophagic vesicles.

To further substantiate the augmented autophagic vesicle formation elicited by Cur in HSFs, a Lyso-tracker fluorescent probe was employed. As shown in Fig. 2H, pronounced red fluorescence signals were observed in HSFs incubated with Cur for either 4 or 24 h, indicative of an accumulation of autophagic vesicles. As with the MDC staining results, the red fluorescence remained prominent and exhibited slight enhancement in the Cur + Rapa and Cur + CQ groups, indicating a reinforced effect. Following co-treatment with 3-MA, the fluorescence intensity was diminished in comparison to the other three groups, with the exception of the control, particularly at the 4 h mark.

#### 3.3.2 mCherry-eRFP-LC3 plasmid transfection

**Figure 3:**
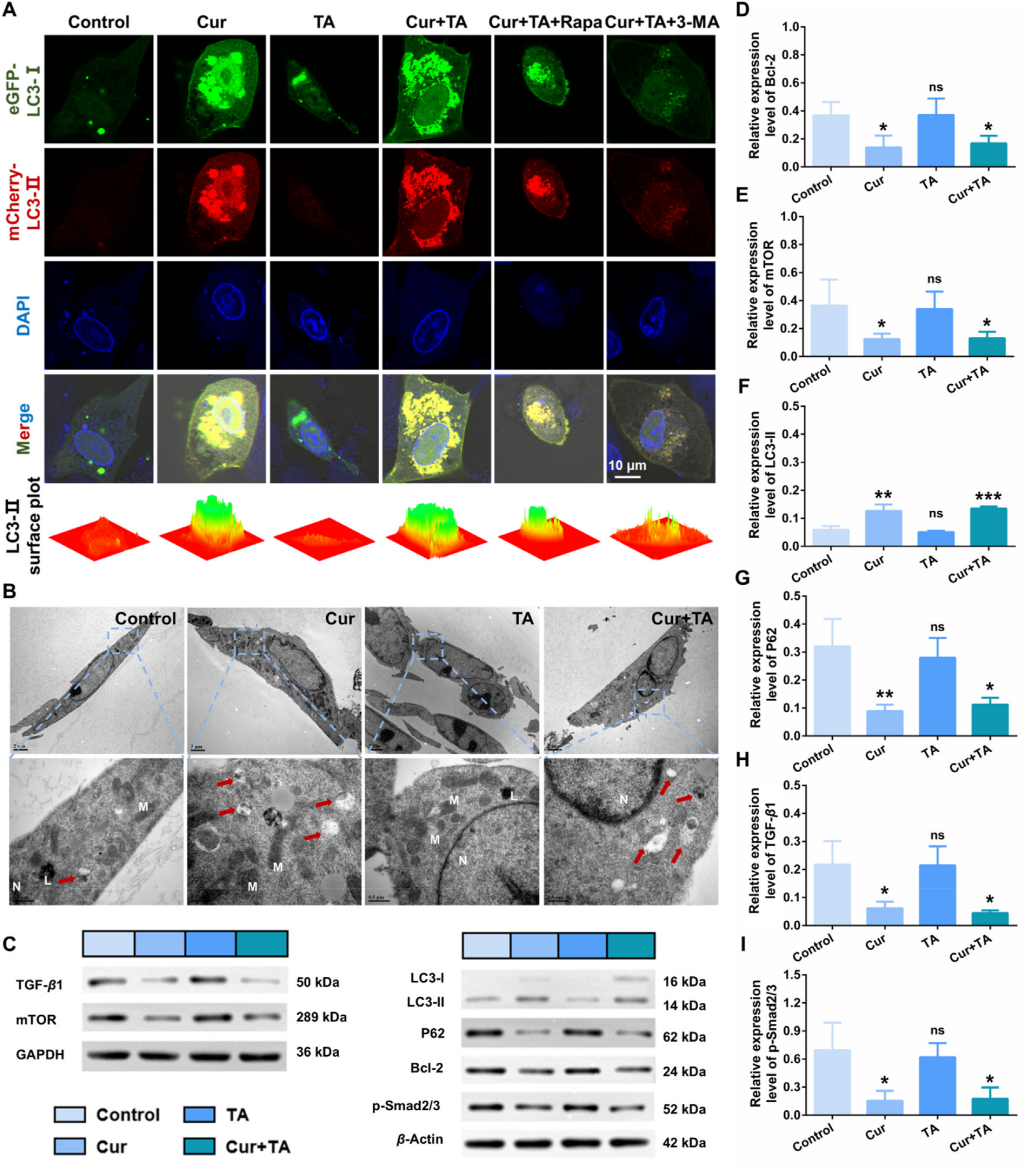
Autophagy level of HSFs after different treatments. (A) Expression of LC3-I and LC3-II in transfected HSFs after different treatments, and the corresponding LC3-II surface plots (scale bar: 10 μm). (B) TEM images of autophagic vacuoles in HSFs after different treatments (scale bar: upper 2 μm and lower 0.5 μm). The red arrows indicate the autophagic vacuoles (N: nucleus, M: mitochondrion, L: lysosome). (C) Western blot analysis of related protein expressions in HSFs after various treatments. (D–I) Statistical analysis of related protein expression levels in HSFs (*n* = 3). Note: ns signified no significant difference *vs* control, ∗*P* < 0.05 *vs* control, ∗∗*P* < 0.01 *vs* control, ∗∗∗*P* < 0.001 *vs* control.

LC3 proteins play a role in the formation of autophagosome membranes, including the conversion of LC3-I to LC3-II. The expression of LC3-I (labelled by eGFP, green fluorescent) and LC3-II (labelled by mCherry, red fluorescent) in HSFs was monitored after different treatments. As shown in Fig. 3A, minimal fluorescence was observed in the control group. The number of fluorescence dots in HSFs were evidently increased after 24 h of Cur incubation. Furthermore, the number of red spots was found to exceed that of green spots, indicating an increased autolysosomes formation and an upregulation of the autophagy level. The TA-treated group exhibited results similar to the control group, indicating that TA had no significant impact on the autophagy process. The combined treatment of Cur and TA demonstrated that TA did not affect the autophagy effect of Cur on HSFs. After the addition of Rapa, the autophagy level was comparable to that of the Cur + TA group, while the number of mCherry-LC3-II red spots was significantly reduced following combination with 3-MA. The findings were consistent with the semi-quantitative results (Supporting Information Fig. S3), indicates the ability of Cur to effectively induce autophagy in HSFs.

#### 3.3.3 Observation of autophagic vacuoles

Autophagosomes present as vacuole-like structures with bilayer or multilayer membranes, containing components such as cytoplasm or damaged organelles^29^. Autolysosomes are defined by the presence of a monolayer membrane and the degradation of cytoplasmic components. In this study, biological TEM was utilized to observe the autophagic vacuoles (including autophagosomes and autolysosomes) in HSFs, and the results are presented in Fig. 3B. In comparison to the control group, the number of autophagic vacuoles in the TA-treated group exhibited minimal change, whereas a notable increase was observed in the groups treated with Cur and Cur + TA (as indicated by the red arrows). These results further substantiate the hypothesis that Cur can enhance the autophagy level of HSFs.

#### 3.3.4 Expression of related proteins

To gain further insight into the function and mechanisms of Cur, a Western blot was used to analyze the expression of signal molecules (Fig. 3C). The anti-apoptotic protein Bcl-2 has been demonstrated to be upregulated in hypertrophic scar tissues^30^. Following treatment with Cur or Cur + TA, Bcl-2 protein levels were found to be significantly downregulated (Fig. 3D). The mTOR protein has been identified as a positive regulator of COL I expression in dermal fibroblasts^31^, while the associated PI3K–AKT–mTOR signaling pathway has been demonstrated to function as a negative regulator of autophagy levels. Elevated expression of the mTOR protein has been reported in scar tissue^32^. As shown in Fig. 3E, the level of mTOR protein was downregulated following treatment with Cur or Cur + TA, which contributed to a reduction in the expression of COL I and the reversal of the inhibition of autophagy. Besides, the expression of the autophagy marker protein LC3 was increased (Fig. 3F), while the expression level of the downstream P62 protein was decreased due to its degradation in autolysosomes (Fig. 3G). As a principal fibrogenic factor, TGF-*β*1 exerts its biological effects primarily through the Smad family of proteins, with phosphorylated Smad2 and Smad3 participating in the transduction of the TGF-*β* signal. This signaling pathway is frequently observed to exhibit sustained aberrant activation in hypertrophic scar tissues^33^. Fig. 3H and I showed that the Cur and Cur + TA groups demonstrated the ability to downregulate the expression level of TGF-*β*1 and the phosphorylation level of downstream Smad2/3 in the scar microenvironment.

The aforementioned results demonstrated that the mechanisms of Cur to induce HSF cell death and suppress hypertrophic scar formation are primarily as follows: (1) promoting of apoptosis, which involves downregulating the expression of Bcl-2 in scar tissues and reversing the blocked apoptosis process; (2) upregulation of autophagy, downregulation of the mTOR signaling pathway, promotion of LC3-Ⅱ expression and P62 degradation, and finally induction of cellular autophagy; (3) downregulation of the TGF-*β* pathway, inhibition of the TGF-*β*1/Smad signaling pathway transduction and ECM production.

### 3.4 Preparation and characterization of MNs

#### 3.4.1 Morphology and element analysis

**Figure 4:**
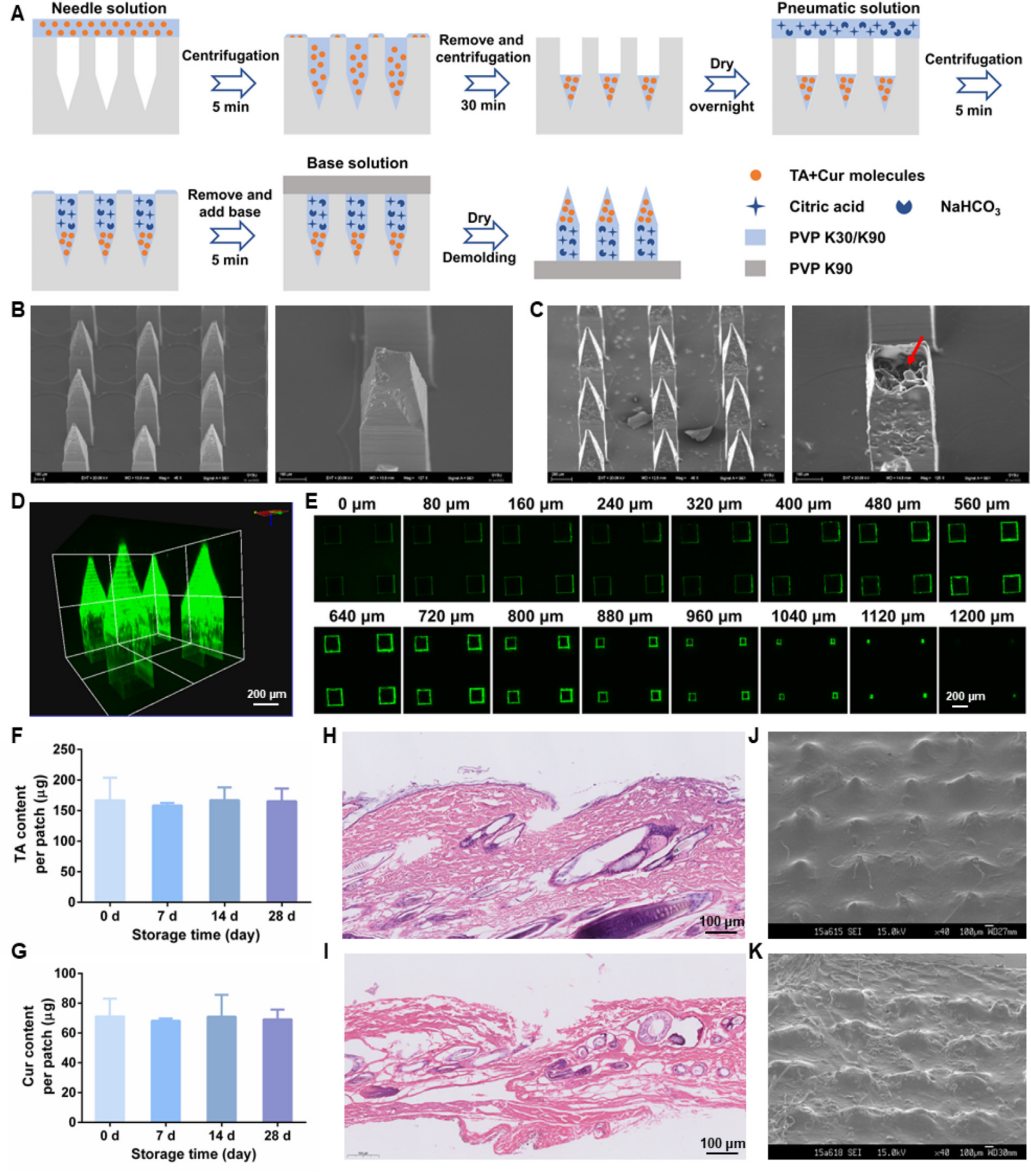
Preparation and characterization of active MNs. (A) Schematic illustration of the preparation of TA-Cur active MNs. SEM images of TA-Cur loaded (B) passive MNs and (C) active MNs (scale bar: left 200 μm, right 100 μm). (D) 3D fluorescence reconstruction (scale bar: 200 μm) and (E) layer-by-layer fluorescence images of TA-Cur active MNs (scale bar: 200 μm). The loading amount of (F) TA and (G) Cur in TA-Cur active MNs and their storage stability at room temperature under dry conditions for 28 days (*n* = 5). H&E-stained sections of inserted rat skin corresponding to (H) passive MNs and (I) active MNs (scale bar: 100 μm). SEM images of the residual (J) passive MNs and (K) active MNs after rat skin insertion for 2 min (scale bar: 100 μm).

The MNs were prepared by a multi-step centrifugal method (Fig. 4A), with a height of 1200 μmol/L and a width of 300 μmol/L arranged in 12 × 12 arrays (Supporting Information Fig. S4). As illustrated in Fig. 4B and C, the overall shape of the active MNs was found to be consistent with that of passive MNs. In the cross-section of the active MNs, the presence of particles (indicated by the red arrow) suggests the incorporation of pneumatic compositions. An EDX analysis was conducted to determine the element distribution, as shown in Supporting Information Fig. S5. In the front view and top view, the presence of substantial C, O, and N elements was evident, accompanied by a ring of Na elements, which indicated the successful loading of sodium bicarbonate in the pneumatic layer. As illustrated in Fig. 4D and E, the fluorescence property of Cur was predominantly deposited on the needle tips, which facilitated the targeted delivery of payloads to the local lesion tissues. The MNs contained 166.8 μg of TA (Fig. 4F) and 71.2 μg of Cur (Fig. 4G) per patch. The drug contents were found to be essentially stable during a 28-day storage period, indicating that the MN system is capable of maintaining the stability of payloads.

#### 3.4.2 Skin puncture ability

It is a prerequisite for effective transdermal drug delivery that the skin be successfully punctured *via* MNs. Firstly, trypan blue-labeled MNs were pressed on isolated rat skin for 2 min, resulting in the formation of an orderly array of micropores, with a puncture rate exceeding 95% (Supporting Information Fig. S6). The H&E staining sections demonstrated that the prepared MNs effectively created microchannels in the skin for drug delivery (Fig. 4H and I). Subsequently, the microneedle patches were collected for observation of dissolution following penetration of the skin. As illustrated in Fig. 4J and K, the needles on the patch backing were completely dissolved without any residues, indicating that the MNs exhibited rapid dissolution in the skin, thereby releasing the pre-loaded drugs.

#### 3.4.3 Gas generation capacity

**Figure 5:**
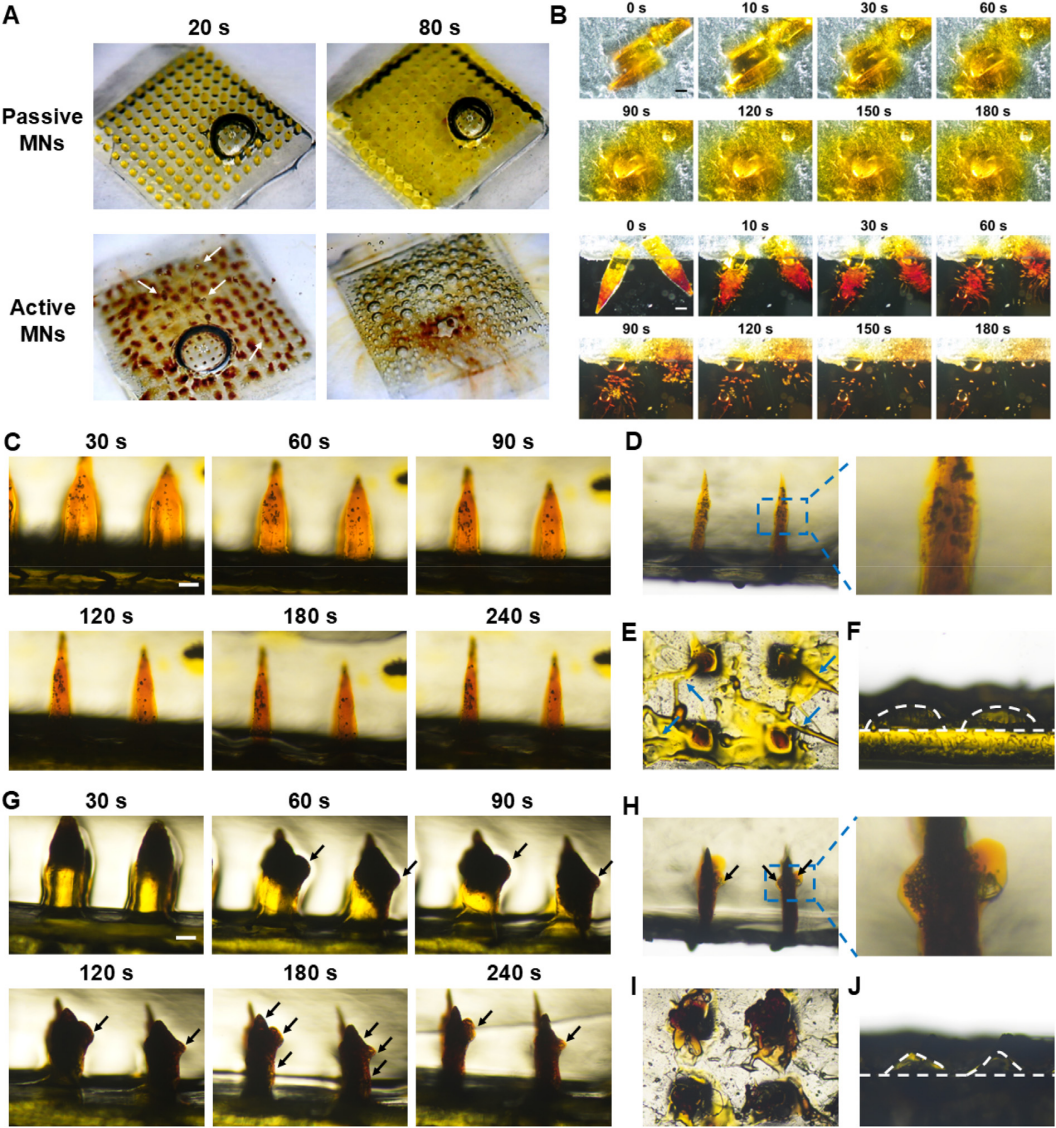
Gas generation capacity of active MNs. (A, B) Gas production capacity of passive MNs and active MNs patch. The white arrows indicate the presence of bubbles. The changes of (C) passive MNs and (G) active MNs in gelatin gel within 4 min. The pictures of MNs accumulated in the gelatin after the removal of (D) passive MNs and (H) active MNs patch. The surface of the gelatin gel after the removal of (E) passive MNs and (I) active MNs. The pictures of dissolved (F) passive MNs and (J) active MNs after being removed from gelatin (scale: 200 μm). The black arrows indicate the presence of bubbles.

To investigate the gas generation capacity of the active MNs, the whole MN patch was exposed to aqueous solutions (Fig. 5A). The passive MNs exhibited minimal dissolution, whereas the active MNs rapidly generated copious bubbles^34^^,^^35^. Besides, the tips of the MNs were cut off to facilitate visualization of the details (Fig. 5B). The passive MNs were observed to undergo continuous dissolution within 180 s after contacting the aqueous solution. In contrast, the tips of the active MNs were able to promptly produce microbubbles within a few seconds. The reaction equation of its pneumatic compositions was as follows, during which abundant CO_2_ microbubbles could be generated^36^.

3NaHCO_3_+C_6_H_8_O_7_=C_6_H_5_O_7_Na_3_+3H_2_O+3CO_2_↑

#### 3.4.4 Dissolution in gelatin-simulated skin

Gelatin blocks with a 35% water content were prepared to simulate skin^37^. As shown in Fig. 5C and G, the passive MNs exhibited a gradual dissolution, whereas the active MNs demonstrated a continuous production of a substantial number of bubbles (indicated by black arrows), demonstrating their superior capacity for gas generation and dissolution in the simulated skin. The patches were removed from the gelatin 4 min later, and the resulting residues were observed. As shown in Fig. 5D and H, the pore channels formed by both MNs could still be discerned within the gelatin blocks. Additionally, the formation of substantial microbubbles by active MNs was discernible upon examination of the magnified view (Fig. 5H). Fig. 5E and I showed the appearance of the gelatin blocks following the removal of MNs. The removal of the passive MNs resulted in the residual adherence of certain drug or patch backing components to the gelatin surface (indicated by the blue arrows in Fig. 5E). In contrast, no residues remained on the gelatin surface for active MNs, which was attributed to the pneumatic layer structure, which accelerates the rapid dissolution. Fig. 5F and J illustrate the appearance of the withdrawn patches following insertion, and both needle bodies were basically dissolved in the gelatin blocks. The above results demonstrate that active MNs present superior dissolution performance and that the generated microbubbles facilitate deep drug penetration.

#### 3.4.5 Drug diffusion and penetration

**Figure 6:**
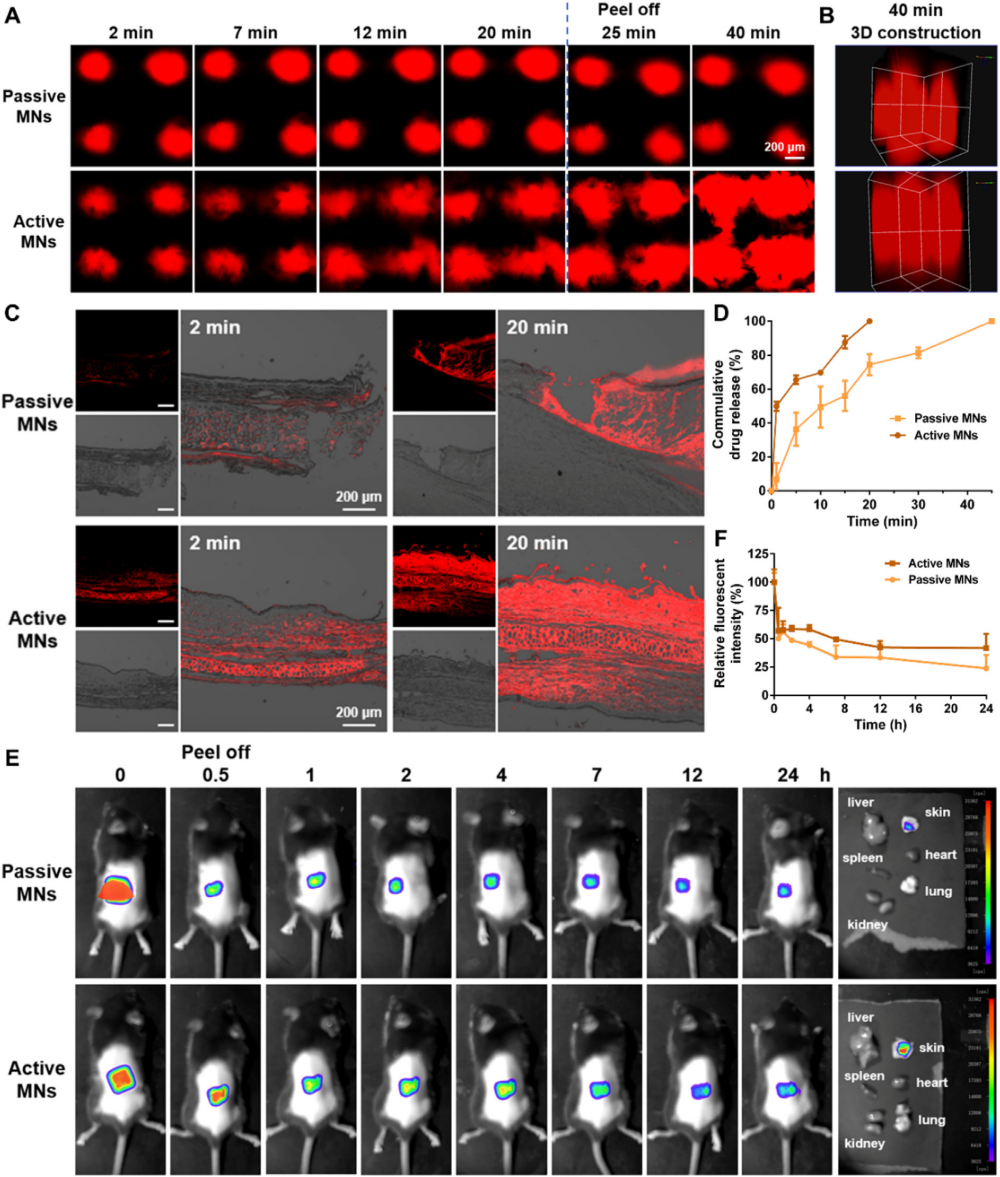
Drug penetration and distribution of active MNs. (A) Drug transverse diffusion and (B) longitudinal penetration in isolated rat skin (scale bar: 200 μm). (C) Drug transverse diffusion and longitudinal penetration in *ex vivo* scar tissue (scale bar: 200 μm). (D) The *in vitro* cumulative drug release profiles of MNs (*n* = 3). (E) *In vivo* fluorescence imaging of mice following administration of MNs. (F) Relative fluorescence intensity over time in the local skin (*n* = 3).

Given that the fluorescence of Cur itself can be readily bleached, C6 and Cy5.5-labeled MNs were prepared for subsequent studies (Supporting Information Fig. S7). As illustrated in Fig. 6A, the transverse drug diffusion of active MNs in isolated rat skin was significantly faster than that of passive MNs. Upon removal of the patch at the 20-min mark, it was observed that the fluorescent drugs delivered by active MNs could continue to diffuse, with full cross-sectional coverage nearly achieved at 40 min. Besides, the 3D reconstruction image revealed that the longitudinal penetration depths of passive MNs and active MNs were 1400 and 2000 μmol/L, respectively (Fig. 6B). Subsequently, the diffusion behavior of MNs in an isolated hypertrophic scar was monitored. The results displayed that the fluorescence intensity was relatively weak at 2 min and 20 min following the puncture of passive MNs (Fig. 6C). In contrast, the active MNs exhibited a strong fluorescence intensity at 2 min, with substantial penetration into the skin dermis at 20 min, displaying uniform and extensive distribution. The 10.13039/501100007874CLSM images also demonstrated that the diffusion of active MNs had reached the outer edge of the scar at 20 min (Supporting Information Fig. S8). These findings indicate that active MNs can effectively improve the transverse diffusion and longitudinal penetration of drugs, thereby achieving a three-dimensional uniform distribution in the scarred lesions. This effect is similar to previously reported hydrogen-powered MNs, which also demonstrated enhanced drug diffusion and penetration in skin tissues through gas-driven propulsion^38^.

#### 3.4.6 *In vitro* drug release

The *in vitro* drug release profiles of TA-Cur loaded MNs are illustrated in Fig. 6D. The passive MNs exhibited complete drug release at 45 min, with about 50% release at 10 min. In contrast, the active MNs demonstrated complete drug release at 20 min. This accelerated release can be attributed to the pneumatic layer, which generated bubbles quickly upon contact with the release medium. This behavior is analogous to that of a pore-forming agent, facilitating the penetration of the release medium into the interior of the MNs and accelerating the disintegration process, thereby promoting the drug release.

#### 3.4.7 *In vivo* drug retention and distribution

A small animal imager was used to monitor the fluorescence intensity on the skin surface. As depicted in Fig. 6E, the initial signal intensity of active MNs was greater than that of passive MNs, indicating that the incorporation of the pneumatic design accelerated the dissolution and drug release of the MNs, which resulted in more drugs being delivered to the body. The maximum local retention in the skin was achieved at 0.5 h (Fig. 6F and Supporting Information Fig. S9), and the fluorescence signal subsequently decreased with time (41.8% after 24 h). In comparison, the passive MNs exhibited a maximum drug release at 1 h, accompanied by a comparatively weaker overall fluorescence intensity (23.9% after 24 h). Then, the main organs and local skin were collected for analysis, which revealed that both MNs did not accumulate in the main organs, thereby avoiding the potential for systemic toxicity. The local skin retention of active MNs was stronger than that of passive MNs. The above results substantiate the assertion that active MNs markedly extended the duration of drug retention, a consequence of the incorporation of pneumatic components.

### 3.5 Inhibition of hypertrophic scar *in vivo*

**Figure 7:**
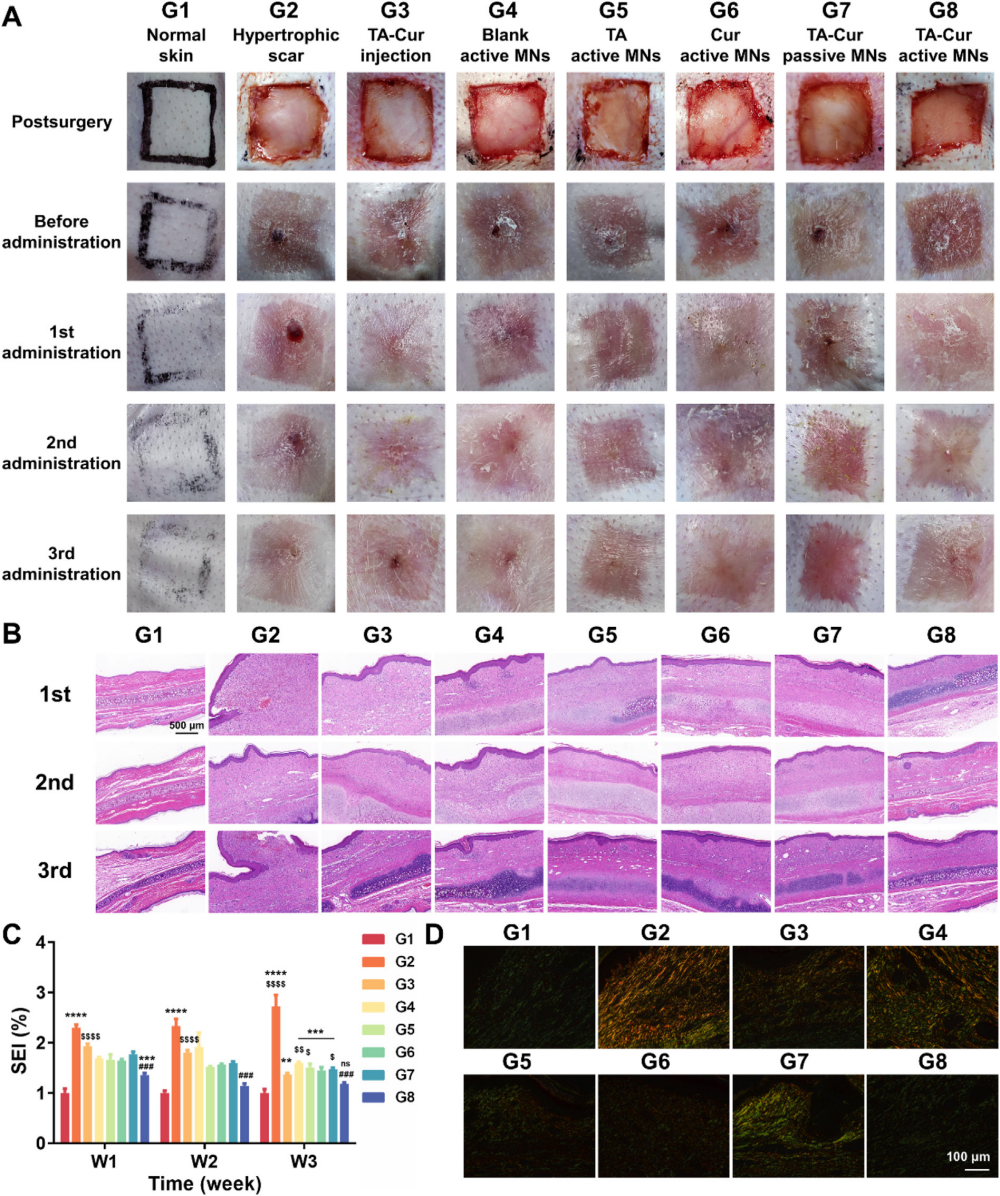
The therapeutic effect of active MNs on rabbit hypertrophic scar model. (A) The appearance of rabbit hypertrophic scar before and after various treatments. (B) H&E-stained histological images (scale bar: 500 μm) and (C) SEI values (*n* = 3) of hypertrophic scar. Note: ns signified no significant difference *vs* normal skin, ∗∗*P* < 0.01 *vs* normal skin, ∗∗∗*P* < 0.001 *vs* normal skin, ∗∗∗∗*P* < 0.001 *vs* normal skin, ^###^*P* < 0.001 *vs* hypertrophic scar, ^$^*P* < 0.05 *vs* TA-Cur active MNs, ^$$^*P* < 0.01 *vs* TA-Cur active MNs, ^$$$$^*P* < 0.0001 *vs* TA-Cur active MNs. (D) Sirius red staining of hypertrophic scar after different treatments (scale bar: 100 μm).

The ear wound sites of New Zealand rabbits were monitored following scar modeling (Fig. 7A). Three weeks post-surgery, the wound had fully epithelialized, the skin texture was hard when touched, and fleshy granular tissues were observed in the center of the wounds. The established hypertrophic scars were randomly grouped for subsequent drug administration.

The rabbits in the normal skin group (G1) were not subjected to any intervention. The hypertrophic scars in the model groups were treated with various preparations. Fig. 7A illustrates that one week after administration, the scar skin in the TA-Cur injection (G3) and TA-Cur active MNs (G8) groups exhibited a reduction in redness and a decrease in thickness. The negative control group (hypertrophic scar, G2) exhibited persistent redness of the skin and bulging fleshy tissues. Following three administrations, the appearance of hypertrophic scar skin showed improvement to a certain extent in all MNs-treated groups and the positive control (G3–G8), particularly in the TA-Cur active MNs group (G8), where the scarred skin was softest to the touch and exhibited the greatest reduction in thickness. The primary rationale was that the use of active MNs facilitated the diffusion and penetration of the drug in dense scar tissues. Additionally, the application of blank active MNs (G4) exhibited a certain improvement effect on skin redness, but the flesh granules can still be observed. In the groups treated with TA active MNs (G5) and Cur active MNs (G6), the redness and swelling were also relieved after treatment. In contrast, the skin in the TA-Cur passive MNs group (G7) exhibited residual redness with distinct borders, primarily due to the dense stromal microenvironment of hypertrophic scars, which impedes diffusion and deep penetration of drugs. Following the third administration of TA-Cur injection, the fleshy granular tissue was observed to persist, and the local lesioned skin exhibited atrophy. These phenomena were attributed to the leakage of the drug from the injection sites and an uneven distribution within the lesions, which led to the occurrence of side effects. The above results substantiate that the TA-Cur active MNs were the most efficacious treatment for hypertrophic scar.

To further evaluate the therapeutic effects, the scar tissue samples were collected one week after each administration for H&E staining analysis to observe the skin structural changes. As illustrated in Fig. 7B, the thickness of the epidermis and dermis layers of the normal rabbit ear exhibited a thickness of approximately 30–70 and 200–600 μmol/L, respectively. The fibroblasts were primarily distributed throughout the dermis, and the collagen fibers exhibited a parallel arrangement with a relatively loose density. In contrast, the hypertrophic scar exhibited a notable thickening of the epidermal layer devoid of discernible patterns, accompanied by an aberrant increase in dermal thickness reaching 1000–2400 μmol/L. Additionally, an increase in the number of abnormally proliferating fibroblasts was observed in the dermis, accompanied by a disorderly arrangement and increased intensity of collagen fibers in nodular or vortex-like distribution. The dermal thickness was observed to decrease to a range of 700–1200 μmol/L following the administration of TA-Cur injection. Following the administration of three doses of blank or single drug-loaded active MNs and TA-Cur passive MNs, a degree of remission of hyperplasia and fibroblast proliferation was observed. Most notably, following the third administration of the TA-Cur active MNs patch, there was a notable reduction in fibroblast and collagen fiber density within the scar tissues, accompanied by an expansion of intercellular space and a notable thinning of the overall skin structure, which approached that observed in the normal skin group. These findings substantiate the efficacy of TA-Cur active MNs in effectively inhibiting fibroblast proliferation and collagen deposition.

SEI allows for a more intuitive quantification of anti-scarring effects, with a smaller SEI indicating a greater efficacy. As illustrated in Fig. 7C, the SEI values following the administration of one or three doses of TA-Cur active MNs were approximately 1.35 and 1.18, respectively. In comparison, the SEI values of the negative control group (G2) were 2.11 and 2.72, respectively. Additionally, the SEI value of 1.18 in G8 following the third administration was the lowest among all treatment groups and exhibited no statistically significant differences compared to the normal skin. Moreover, during the first two administrations, the SEI values of the TA-Cur active MNs group were significantly lower than those of the TA-Cur injection group. However, the difference between the two groups was not significant after the third administration. We speculate that this is because the scar tissue had largely recovered by the third administration, bringing the SEI values of both groups closer to normal levels. This result also highlights that the active MNs exert a rapid therapeutic effect during the early treatment phase. It is noteworthy that G4 (blank active MNs) also demonstrated efficacy in relieving scar hyperplasia compared to G2, with an SEI value of 1.60 after three doses. This effect can be attributed to the arrayed longitudinal tangential forces generated by multi-point insertion of MNs, which endow the ability to repair and regulate the scar stromal microenvironment.

The scar tissue samples were subjected to Masson staining analysis to observe any changes in the tissue collagen fiber. Following Masson staining, the epidermis layer exhibited a light purple hue, while the upper epidermis displayed a dark purple coloration. The collagen fibers, mucus, and cartilage appeared blue, and the muscle fibers, cytoplasm, and muscle were observed to be red. As shown in Supporting Information Fig. S10, the rabbit ear tissues in the normal skin group exhibited a low density and an orderly arrangement of collagen fibers. However, a considerable number of collagen fibers in the hypertrophic scar group were observed to be misarranged, exhibiting varying thickness and dense accumulation, and some crossed into nodular or vortex-like distributions. All groups exhibited a reduction in collagen fiber density to varying degrees. Particularly, the TA-Cur active MNs exhibited the most pronounced improvement effect. The arrangement of collagen fiber became increasingly regular, predominantly parallel to the epidermal layer, and nodular or vortex-like deposition was no longer observed.

Sirius red is a strongly acidic substance that can readily bind to the basic groups present in collagen molecules. Polarized light microscopy reveals that tightly arranged COL I appears red or yellow, whereas COL III appears as green with fine fibers. As illustrated in Fig. 7D, both COL I and COL III were present in the normal rabbit ear tissue, with numerous scattered COL III and occasional COL I. In the scar model group (G2), a substantial quantity of coarse and densely arranged COL I was observed, accompanied by a notable reduction in COL III content. The ratio of COL I/III was significantly elevated in comparison to the normal skin group (G1), indicating that the aberrant COL I synthesis represents a pivotal mechanism underlying scar hyperplasia. Following three administrations, a slight reduction in the amount of COL I was observed in the blank active MNs (G4) and the TA-Cur passive MNs group (G7). The TA-Cur active MNs (G8) exhibited the greatest capacity to inhibit COL I synthesis, approaching the distribution ratio of COL I/III observed in G1. Meanwhile, their efficacy surpassed that of single drug-loaded MNs (G5 and G6), indicating that the combination of TA and Cur was more effective for promoting scar repair.

### 3.6 Protein expression analysis

**Figure 8:**
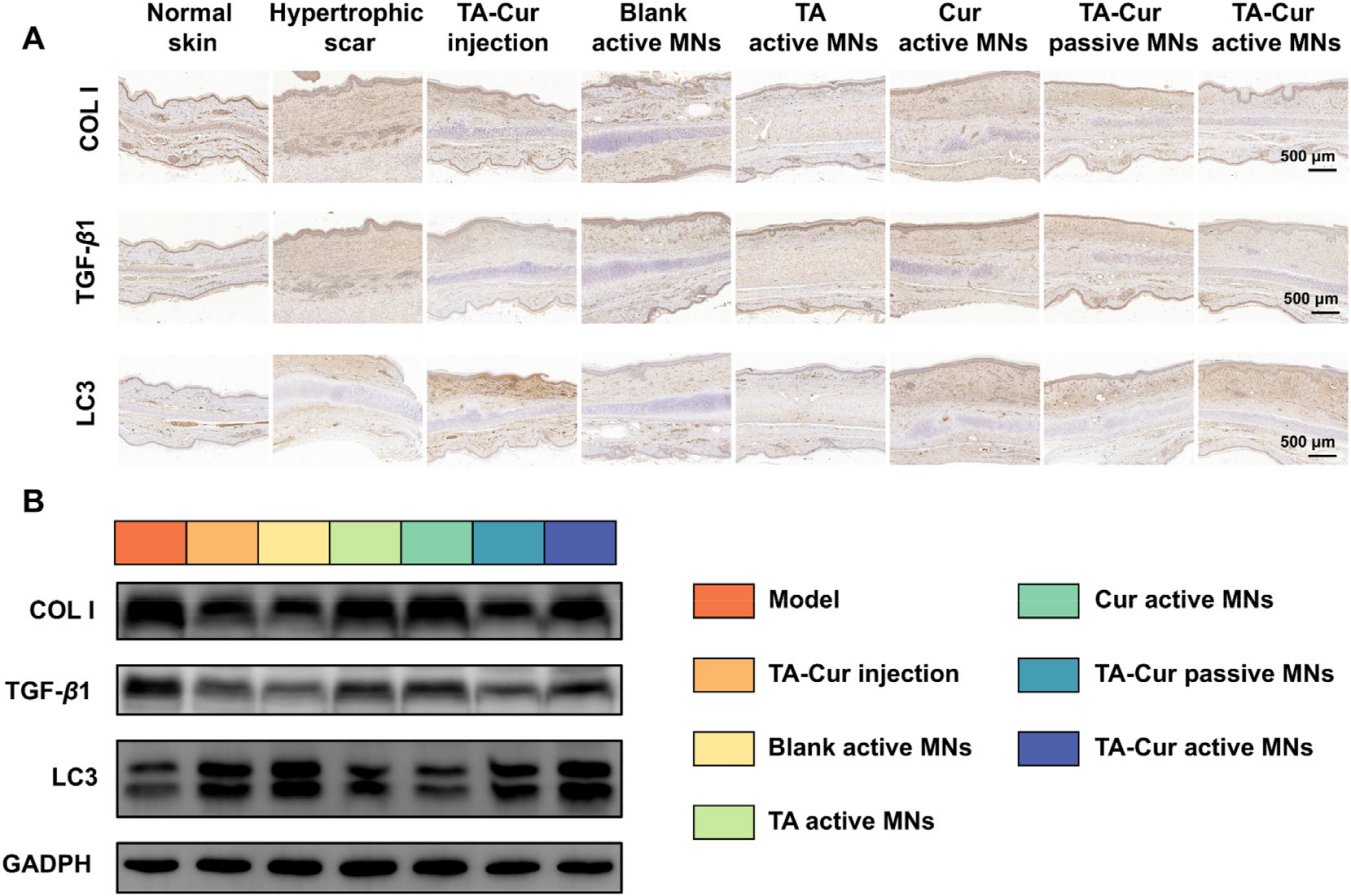
Histological and Western blot analysis of active MNs on rabbit hypertrophic scar model at the end of the intervention. (A) Immunohistochemical staining analysis of Collagen I, TGF-*β*1, and LC3 in hypertrophic scar tissues after various treatments (scale bar: 500 μm). (B) Western blot analysis of related protein expressions in hypertrophic scar tissues after various treatments.

The expression of COL I, TGF-*β*1, and LC3 proteins in rabbit ear tissues was evaluated through IHC staining. As illustrated in Fig. 8A, the elevated brown particle deposition in the dermis of the hypertrophic scar group indicated that the scar development would be accompanied by an increased expression of the COL I and TGF-*β*1 proteins. Notably, in addition to the drug-treated groups, the blank MN also decreased the expression of COL I and TGF-*β*1. This effect was primarily attributed to the physical intervention of MNs, which was capable of regulating local mechanical stress and thereby improving the pathological characteristics of scars^39^. Following treatment with TA-Cur active MNs, the positive staining was diminished, indicating a reduction in the levels of COL I and TGF-*β*1. Similarly, the degree of LC3 positive labeling was enhanced in the Cur-containing groups, indicating that Cur could promote the upregulation of autophagy levels and contribute to induction of cell death in overproliferated fibroblasts.

Western blot analysis was conducted to further investigate the expression of proteins following three administrations. As illustrated in Fig. 8B and Supporting Information Fig. S11, the expression levels of COL I and TGF-*β*1 proteins were elevated in the hypertrophic scar group. The TA-Cur active MNs treatment demonstrated a more pronounced reduction in the expression of these two proteins and an enhanced upregulation of LC3 protein. The results were consistent with the above IHC staining analysis, thereby verifying that TA-Cur active MNs could effectively reduce COL I and TGF-*β*1 expression in hypertrophic scar tissues, avoid excessive deposition of ECM components, promote LC3 protein, and upregulate the autophagy level to reshape the scar microenvironment and achieve the successful repair.

### 3.7 *In vivo* safety evaluation

To observe whether the skin channels caused by MN administration were reversible, a piece of drug-loaded passive MNs or active MNs patch was applied on the back skin of C57BL/6 female mice, and then the skin recovery was observed periodically. As shown in Fig. S12, mice in both groups had obvious puncture pores right after MNs insertion, with slight redness on the skin surface. Then the pores began to close gradually a few hours after administration and basically returned to normal 24 h later, indicating that the MN patches were tolerable and safe. To further evaluate the systemic safety of the active MNs, the main organs (heart, liver, spleen, lung, and kidney) of mice treated with active MNs were collected for H&E staining. The results showed that no pathological damage was observed in the major organs (Fig. S13). Furthermore, the blood samples of mice were collected for complete blood count (CBC) analysis. As shown in Fig. S14, the blood routine parameters did not show significant differences compared to the normal group. The results indicated that the active MN system does not cause adverse systemic effects.

## 4 Conclusions

This study presented an efficacious strategy to reshape the scar microenvironment through the regulation of autophagy for the treatment of hypertrophic scar. This was accomplished through the fabrication of a binary-drug loaded active MNs transdermal system. Upon insertion into the hypertrophic scar tissue, the active MNs rapidly dissolved, prompting the pneumatic components to produce copious amounts of microbubbles. The microbubbles contributed to the weakening of the adhesion force between the needle tips and the patch backing, facilitating their rapid separation. In addition, the generation of copious bubbles was accompanied by vigorous vortex fluid flows, which acted as a propulsive force to drive the autonomous diffusion and penetration of drugs, thereby realizing faster and deeper drug delivery to the dense scar tissue. The released TA and Cur could jointly alleviate the fibrosis level of the hypertrophic scar by upregulating the autophagy level, inducing apoptosis of HSFs, inhibiting collagen synthesis, and downregulating TGF-*β*1 expression, which collectively underscored the therapeutic potential of autophagy induction in treating hypertrophic scars. The developed active MNs system also offers a novel approach to deep drug delivery for combating dense tissues, with significant prospects for biomedical applications.

## Author contributions

Ting Wen: Writing – original draft, Visualization, Methodology, Investigation, Formal analysis. Yanping Fu: Writing – original draft, Visualization, Methodology, Investigation, Formal analysis. Xiangting Yi: Visualization, Methodology, Investigation. Ying Sun: Methodology, Investigation. Wanchen Zhao: Visualization, Investigation. Chaonan Shi: Investigation. Ziyao Chang: Methodology. Beibei Yang: Methodology. Shuling Li: Methodology. Chao Lu: Investigation. Tingting Peng: Investigation. Chuanbin Wu: Project administration, Funding acquisition. Xin Pan: Supervision, Project administration, Investigation, Funding acquisition, Conceptualization. Guilan Quan: Writing – review & editing, Supervision, Project administration, Investigation, Funding acquisition, Conceptualization.

## Conflicts of interest

The authors declare that they have no known competing financial interests or personal relationships that could have appeared to influence the work reported in this paper.

## Appendix A Supporting information

The following are the Supporting Information to this article.
