# Supplementary material for: Autonomous drug delivery and scar microenvironment remodeling using micromotor-driven microneedles for hypertrophic scars therapy
Source: Acta Pharm Sin B. 2025 May 21;15(7):3738–55. doi: 10.1016/j.apsb.2025.05.017 (PMC12278434; doi:10.1016/j.apsb.2025.05.017)
Supplement: Multimedia component 1 [file mmc1.pdf]

Supporting Information for

Original article

Autonomous drug delivery and scar microenvironment remodeling using micromotor-driven microneedles for hypertrophic scars therapy

Ting Wen[a,†], Yanping Fu[b,†], Xiangting Yi[b], Ying Sun[a], Wanchen Zhao[b], Chaonan Shi[b], Ziyao Chang[a], Beibei Yang[a], Shuling Li[b], Chao Lu[b], Tingting Peng[b], Chuanbin Wu[b,c], Xin Pan[a,*], Guilan Quan[b,*]

[a]*School of Pharmaceutical Sciences, Sun Yat-sen University, Guangzhou 510006, China*

[b]*State Key Laboratory of Bioactive Molecules and Druggability Assessment, Guangdong Basic Research Center of Excellence for Natural Bioactive Molecules and Discovery of Innovative Drugs, College of Pharmacy, Jinan University, Guangzhou 510632, China*

[c]*Jiangmen Wuyi Hospital of Traditional Chinese Medicine, Affiliated Jiangmen Traditional Chinese Medicine Hospital of Jinan University, Jiangmen 529031, China*

Received 30 December 2024; received in revised form 7 March 2025; accepted 25 March 2025

[*]Corresponding authors.

E-mail addresses: Xin Pan (panxin2@mail.sysu.edu.cn), Guilan Quan (quanguilan@jnu.edu.cn)

[†]These authors made equal contributions to this work.

Supporting experimental details

1. Materials

Dipotassium phosphate was acquired from Damao Chemical Reagent Factory (Tianjin, China). Monopotassium phosphate was provided by Sinopharm Chemical Reagent Co., Ltd. (Shanghai, China). Chromatographically pure acetonitrile was purchased from Honeywell International Inc (NJ, USA), and glacial acetic acid was obtained from Guangzhou Chemical Reagent Factory (Guangzhou, China). Trypan blue was acquired from Beyotime Biotechnology Co., Ltd. (Shanghai, China). DMEM F-12 medium, fetal bovine serum (FBS), sterile phosphate buffer saline (PBS), 0.25% trypsin-EDTA, and penicillin-streptomycin solution (100×) were all acquired from Gibco Life Technologies (Grand Island, NY, USA). The mCherry-eGFP-LC3 double labeled plasmid was provided by Shanghai GenePharma Co., Ltd. (Shanghai, China), and jetPRIME® transfection reagent was purchased from Polyplus (Illkirch, France). The PAGE gel rapid preparation kit was supplied by Shanghai Epizyme Biotech Co., Ltd. (Shanghai, China). The antibodies of anti-beta actin, anti-GAPDH [6C5], anti-mTOR [Y391], and anti-SQSTM1/p62 were obtained from Abcam Plc (Cambridge, UK). Anti-phospho-Smad2/Smad3 (Thr8), anti-TGF beta 1, and anti-Collagen I antibodies were provided by Bioss Biotech Co., Ltd. (Beijing, China). LC3B antibody and LC3 polyclonal antibody were acquired from Cell Signaling Technology, Inc (Boston, USA) and Proteintech Group Inc (Wuhan, China), respectively. Anti-Bcl-2 antibody was supplied by R&D Systems (MN, USA). HRP-Goat anti Rabbit and HRP-Goat anti Mouse were purchased from Elabscience Biotech Co., Ltd. (Wuhan, China). Ultrapure water (resistivity: 18.2 MΩ·cm) was processed by the Ultra Bio Mk2 ultrapure system (Elga, Marlow, UK).

2. Methods

2.1. Cell culture

HSFs were cultured in DMEM F-12 medium complemented with 10% FBS and 1% penicillin–streptomycin solution and transferred to the 37 °C incubator containing 5% CO_2 overnight. When the cells grew to the integration degree of 80%–90%, 0.25% trypsin was applied to digest the cells for subsequent experiments.

2.2. Cytotoxicity test

HSFs were seeded in 96-well plates at the density of 8×10^3 cells per well, and then cultured in the cell incubator for 24 h. The concentration range of TA was set to be 0–300 μmol/L, and that of Cur was 0–30 μmol/L. After 24 h of incubation, the CCK-8 working solution (110 μL, CCK-8: DMEM

F-12= 1:10 *v/v*) was added to each well. The relative cell viability was calculated by the absorbance at 450 nm recorded using a microplate reader (Synergy H1, BioTek, USA).

2.3. Establishment of drug content determination methodology

High-performance liquid chromatography (HPLC) was applied to determine the contents of TA and Cur. For TA, 20 μL of sample solution was injected with 40% ultrapure water and 60% acetonitrile as mobile phases at a flow rate of 1.0 mL/min, and the ultraviolet (UV) detection wavelength was set as 240 nm. For Cur, 20 μL of sample solution was injected with 42% ultrapure water containing 0.5% glacial acetic acid and 58% acetonitrile as mobile phases at a flow rate of 1.0 mL/min, and the UV detection wavelength was set as 430 nm.

2.4. Payload determination and storage stability

To determine the payload contents of TA-Cur active MNs, the needle tips were completely excised and collected to dissolve in 1 mL of anhydrous ethanol. The contents of TA and Cur encapsulated in MNs were determined by HPLC. Besides, the prepared TA-Cur active MNs were stored in a desiccator at room temperature, and the drug content changes were monitored within 4 weeks to evaluate their storage stability.

Supporting figures

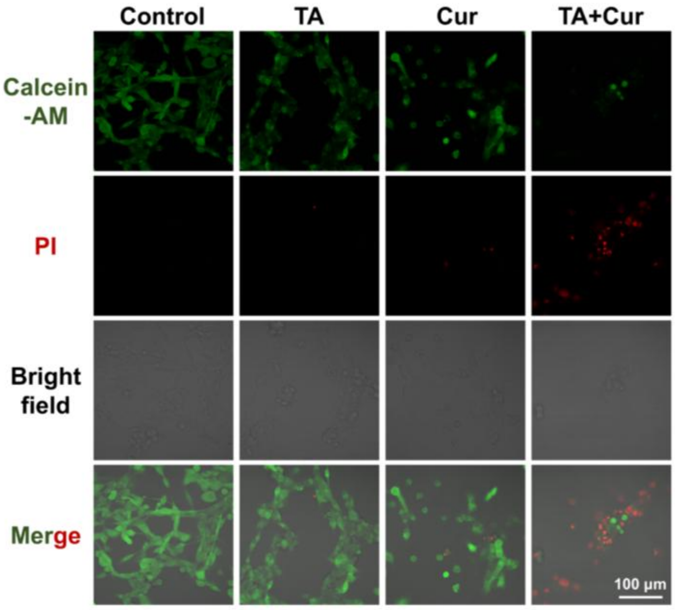

Figure S1 Live/dead cell staining after different treatments (scale bar: 100 μm).

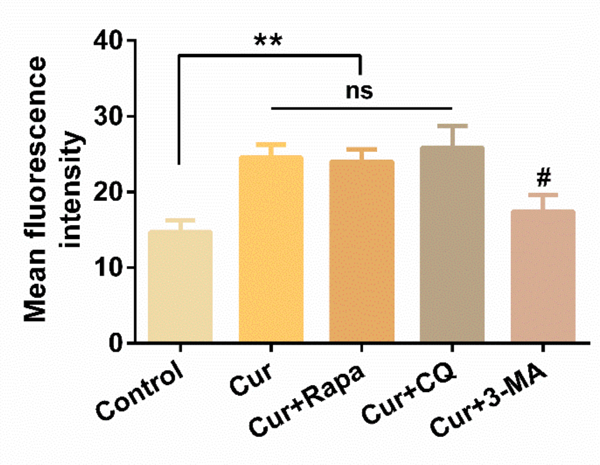

Figure S2 The semi-quantitative result of MDC mean fluorescence intensity in HSF cells after different treatments (n = 3). Note: $^{**}P$<0.01 *vs* Control, $^{\#}P$<0.05 *vs* Cur.

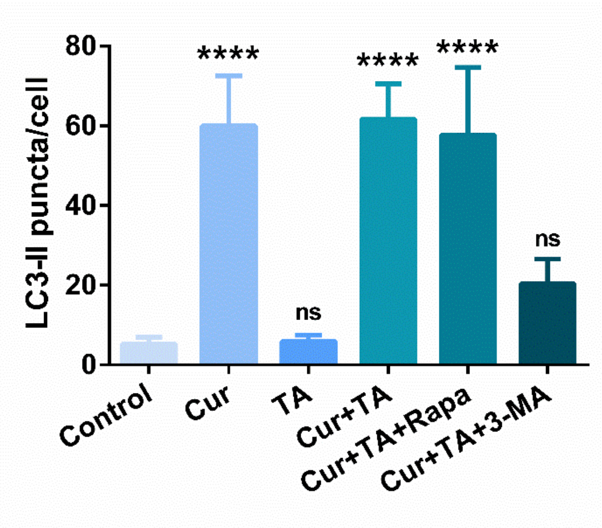

Figure S3 The semi-quantitative result of LC3-II expression in HSF cells after various treatments for 24 h (n = 3). Note: ns signified no significant difference *vs* control, ****P<0.0001 *vs* control.

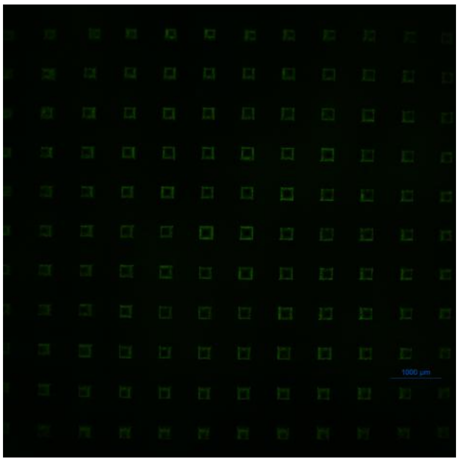

Figure S4 The full fluorescence scanning image of active MNs.

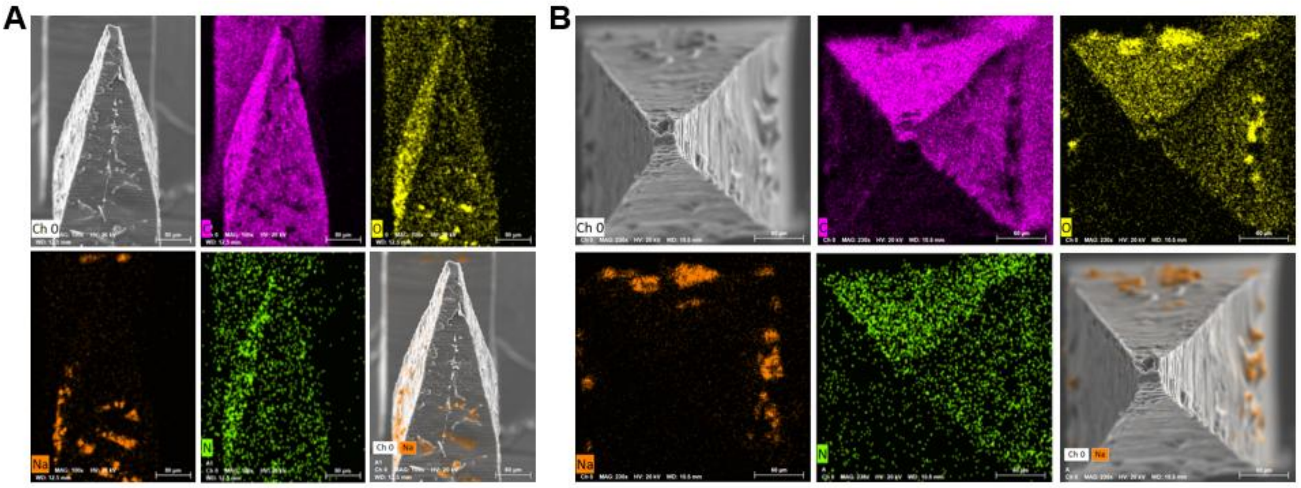

Figure S5 EDX element analysis of dual drug-loaded active MNs in (A) front view and (B) top view.

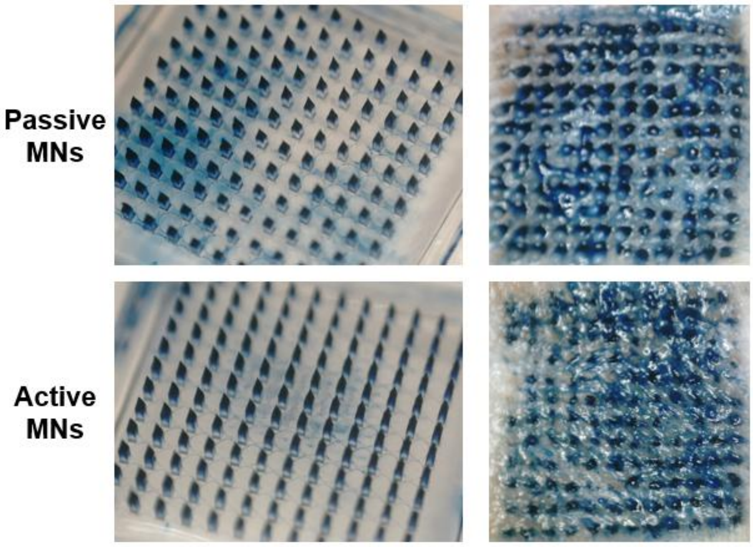

Figure S6 The photographs of trypan blue-loaded passive MNs and active MNs, and the corresponding isolated rat skin after microneedle insertion.

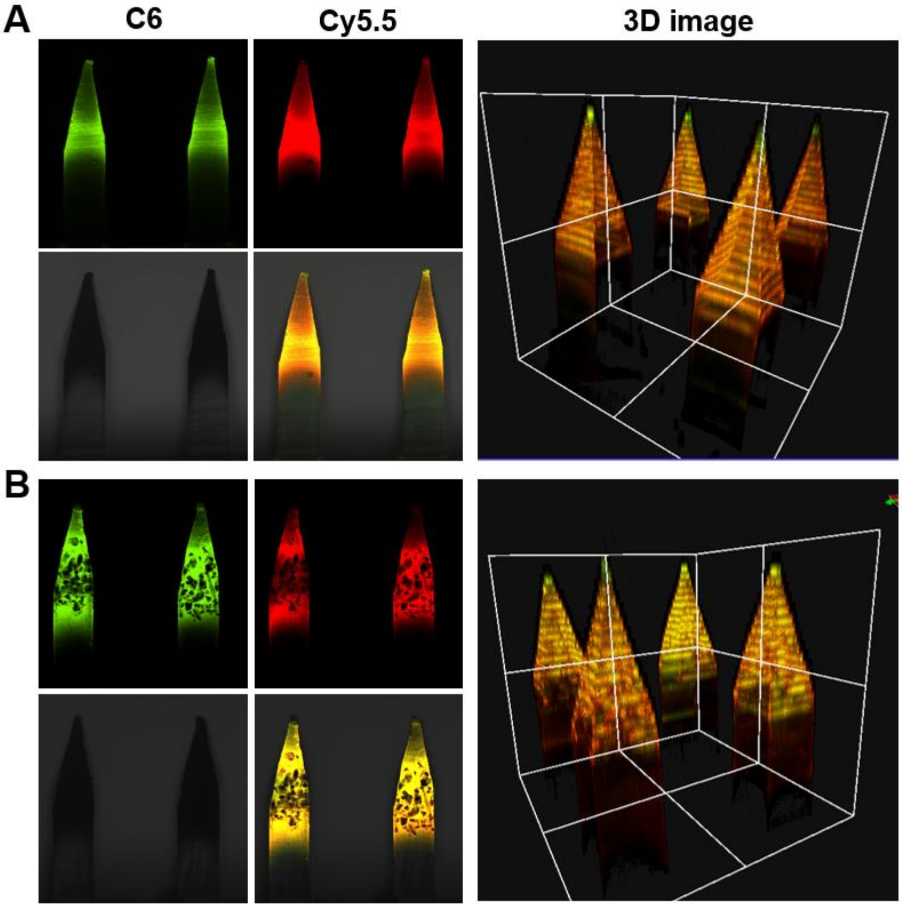

Figure S7 Fluorescence distribution and 3D construction of (A) passive MNs and (B) active MNs.

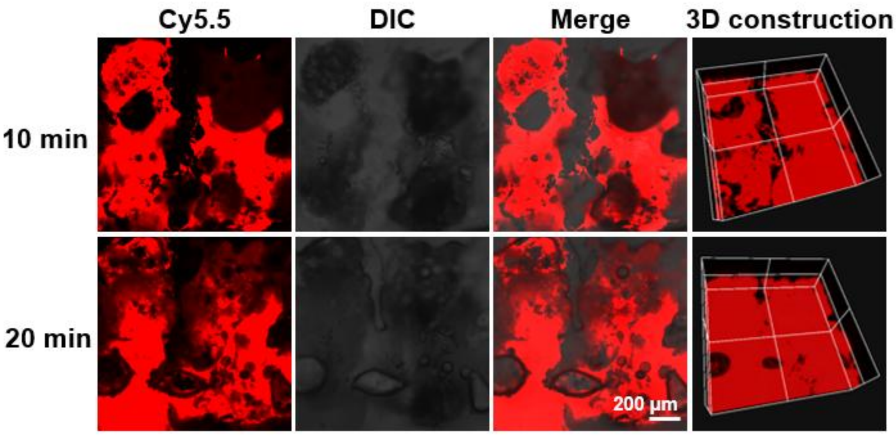

Figure S8 Drug transverse diffusion in *ex vivo* scar tissue for active MNs (scale bar: 200 μm).

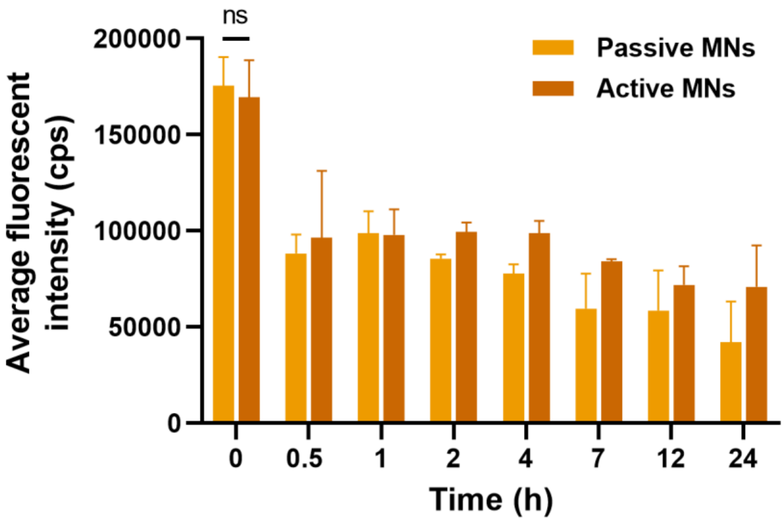

Figure S9 Average fluorescence intensity over time in the local skin of mice (n = 3).

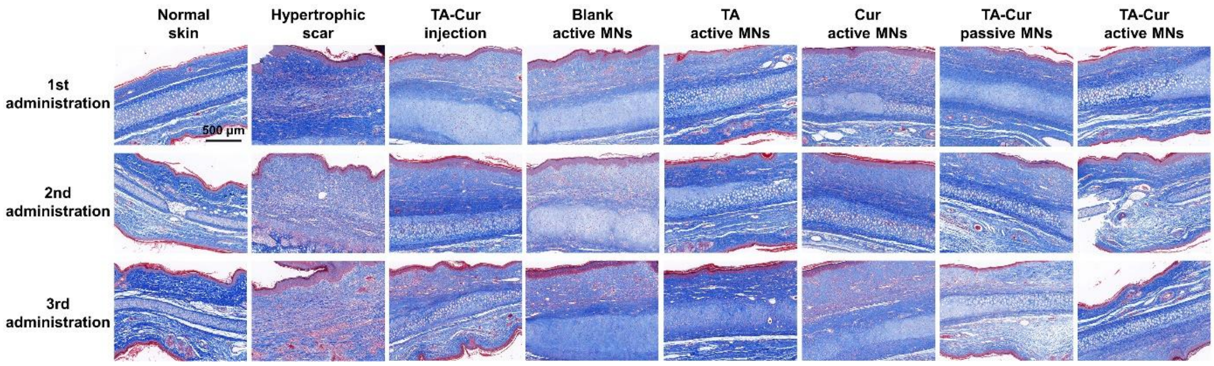

Figure S10 Masson's trichrome staining of hypertrophic scar at different time points after administration (scale bar: 500 μm).

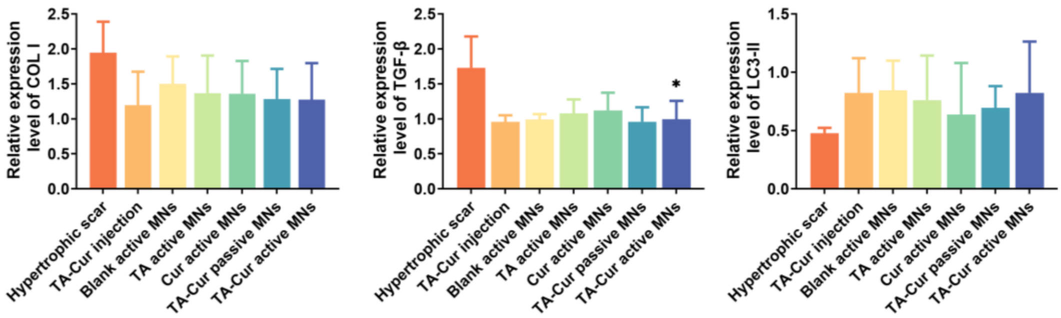

Figure S11 Statistics analysis of related protein expression levels in hypertrophic scar tissues after various treatments ($n = 3$).

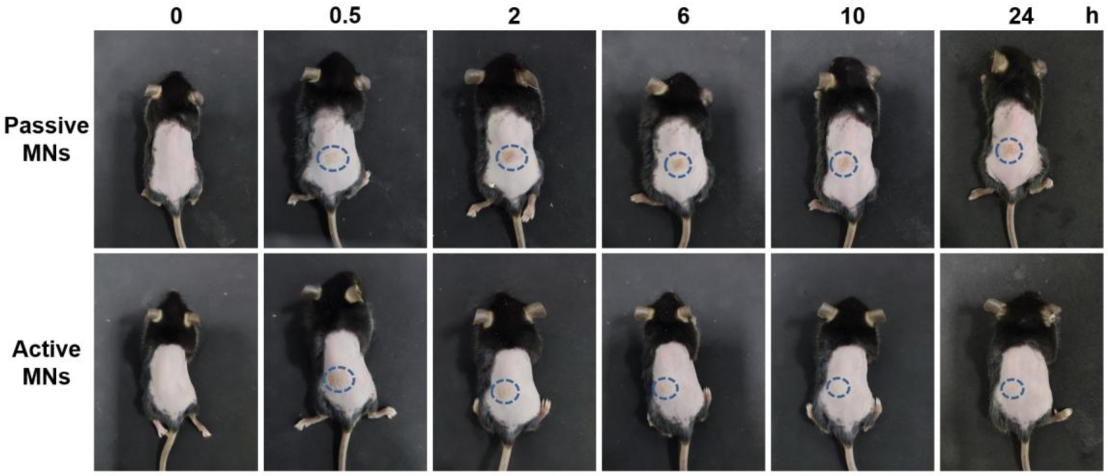

Figure S12 Pictures of mice dorsal skin at predetermined time points after treatment with passive MNs or active MNs.

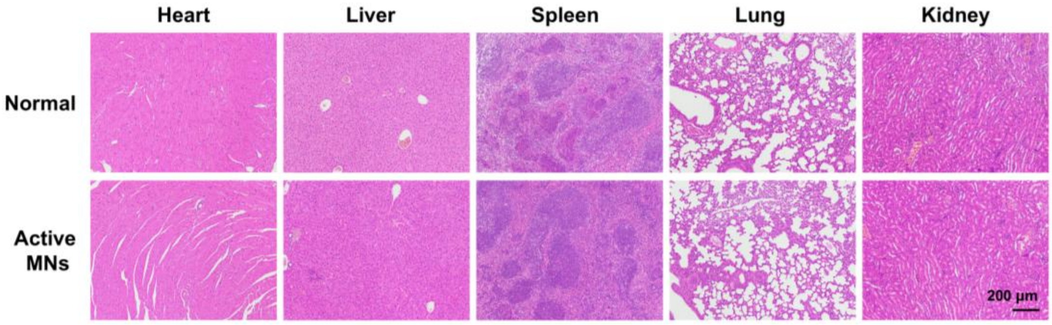

Figure S13 H&E staining images of mice major organs after treatment with active MNs. (Scale bar: 200 μm).

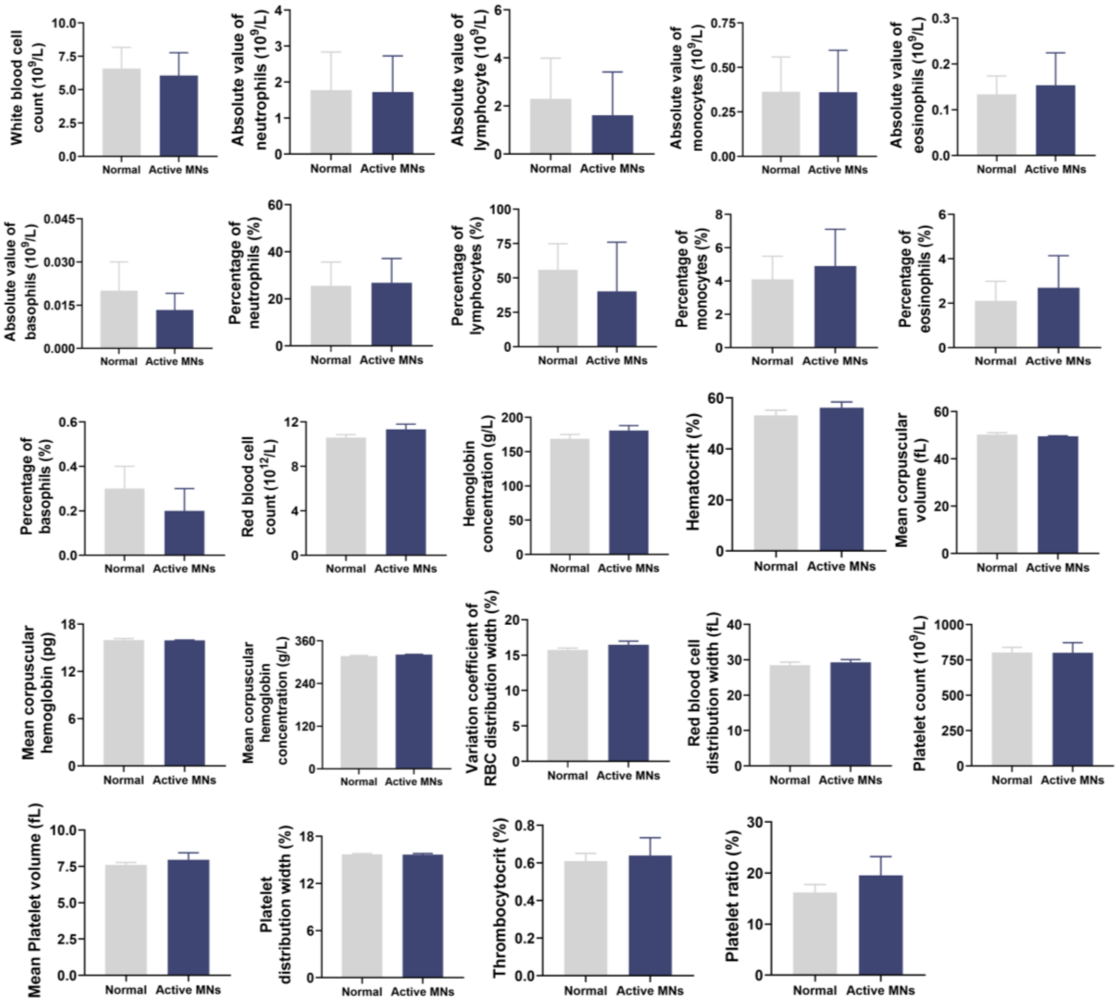

Figure S14 Complete blood count analysis of mice after treatment with active MNs (n = 3).
